# Comparative Tumor RNA Sequencing Analysis for Difficult-to-Treat Pediatric and Young Adult Patients With Cancer

**DOI:** 10.1001/jamanetworkopen.2019.13968

**Published:** 2019-10-25

**Authors:** Olena M. Vaske, Isabel Bjork, Sofie R. Salama, Holly Beale, Avanthi Tayi Shah, Lauren Sanders, Jacob Pfeil, Du L. Lam, Katrina Learned, Ann Durbin, Ellen T. Kephart, Rob Currie, Yulia Newton, Teresa Swatloski, Duncan McColl, John Vivian, Jingchun Zhu, Alex G. Lee, Stanley G. Leung, Aviv Spillinger, Heng-Yi Liu, Winnie S. Liang, Sara A. Byron, Michael E. Berens, Adam C. Resnick, Norman Lacayo, Sheri L. Spunt, Arun Rangaswami, Van Huynh, Lilibeth Torno, Ashley Plant, Ivan Kirov, Keri B. Zabokrtsky, S. Rod Rassekh, Rebecca J. Deyell, Janessa Laskin, Marco A. Marra, Leonard S. Sender, Sabine Mueller, E. Alejandro Sweet-Cordero, Theodore C. Goldstein, David Haussler

**Affiliations:** 1 Department of Molecular, Cell, and Developmental Biology, University of California, Santa Cruz; 2 University of California, Santa Cruz Genomics Institute, Santa Cruz; 3 Howard Hughes Medical Institute, University of California, Santa Cruz; 4 Division of Hematology and Oncology, Department of Pediatrics, University of California, San Francisco; 5 Integrated Cancer Genomics Division, Translational Genomics Research Institute (TGen), Phoenix, Arizona; 6 Cancer and Cell Biology Division, TGen, Phoenix, Arizona; 7 Center for Data Driven Discovery in Biomedicine, Children’s Hospital of Philadelphia, Philadelphia, Pennsylvania; 8 Stanford Cancer Institute, Stanford University School of Medicine, Stanford, California; 9 CHOC Children’s Hospital, Hyundai Cancer Institute, Orange, California; 10 British Columbia Children’s Hospital Research Institute, British Columbia Children’s Hospital, Vancouver, British Columbia, Canada; 11 BC Cancer, Vancouver, British Columbia, Canada; 12 Canada’s Michael Smith Genome Sciences Centre, BC Cancer, Vancouver, British Columbia, Canada; 13 Department of Medical Genetics, University of British Columbia, Vancouver, British Columbia, Canada; 14 Department of Neurology, University of California, San Francisco; 15 Department of Neurosurgery, University of California, San Francisco; 16 Department of Pediatrics, University of California, San Francisco; 17 Now with Anthem, Inc, Palo Alto, California

## Abstract

**Importance:**

Pediatric cancers are epigenetic diseases; therefore, considering tumor gene expression information is necessary for a complete understanding of the tumorigenic processes.

**Objective:**

To evaluate the feasibility and utility of incorporating comparative gene expression information into the precision medicine framework for difficult-to-treat pediatric and young adult patients with cancer.

**Design, Setting, and Participants:**

This cohort study was conducted as a consortium between the University of California, Santa Cruz (UCSC) Treehouse Childhood Cancer Initiative and clinical genomic trials. RNA sequencing (RNA-Seq) data were obtained from the following 4 clinical sites and analyzed at UCSC: British Columbia Children’s Hospital (n = 31), Lucile Packard Children’s Hospital at Stanford University (n = 80), CHOC Children’s Hospital and Hyundai Cancer Institute (n = 46), and the Pacific Pediatric Neuro-Oncology Consortium (n = 24). The study dates were January 1, 2016, to March 22, 2017.

**Exposures:**

Participants underwent tumor RNA-Seq profiling as part of 4 separate clinical trials at partner hospitals. The UCSC either downloaded RNA-Seq data from a partner institution for analysis in the cloud or provided a Docker pipeline that performed the same analysis at a partner institution. The UCSC then compared each participant’s tumor RNA-Seq profile with more than 11 000 uniformly analyzed tumor profiles from pediatric and young adult patients with cancer, downloaded from public data repositories. These comparisons were used to identify genes and pathways that are significantly overexpressed in each patient’s tumor. Results of the UCSC analysis were presented to clinical partners.

**Main Outcomes and Measures:**

Feasibility of a third-party institution (UCSC Treehouse Childhood Cancer Initiative) to obtain tumor RNA-Seq data from patients, conduct comparative analysis, and present analysis results to clinicians; and proportion of patients for whom comparative tumor gene expression analysis provided useful clinical and biological information.

**Results:**

Among 144 samples from children and young adults (median age at diagnosis, 9 years; range, 0-26 years; 72 of 118 [61.0%] male [26 patients sex unknown]) with a relapsed, refractory, or rare cancer treated on precision medicine protocols, RNA-Seq–derived gene expression was potentially useful for 99 of 144 samples (68.8%) compared with DNA mutation information that was potentially useful for only 34 of 74 samples (45.9%).

**Conclusions and Relevance:**

This study’s findings suggest that tumor RNA-Seq comparisons may be feasible and highlight the potential clinical utility of incorporating such comparisons into the clinical genomic interpretation framework for difficult-to-treat pediatric and young adult patients with cancer. The study also highlights for the first time to date the potential clinical utility of harmonized publicly available genomic data sets.

## Key Points

**Question:**

Is it feasible and useful to compare the tumor RNA sequencing data of a child or young adult with the tumor RNA sequencing data of thousands of other patients (of all ages) in a research setting?

**Findings:**

Among 144 tumor samples from children and young adults, comparative RNA sequencing analysis, conducted across 4 precision medicine studies in the United States and Canada, was feasible and potentially useful for 99 of 144 pediatric and young adult cancer samples. In contrast, DNA mutation information was potentially useful for only 34 of 74 samples.

**Meaning:**

This study’s findings suggest that open sharing and combined analysis of tumor RNA sequencing data from pediatric and young adult patients treated on different clinical trials may represent a feasible approach and may produce useful clinical and biological information for individual patients.

## Teaser

This cohort study evaluates the feasibility and utility of incorporating comparative gene expression information into the precision medicine framework for difficult-to-treat pediatric and young adult patients with cancer.

## Introduction

We present a framework for comparative RNA sequencing (RNA-Seq) analysis of pediatric tumors across multiple precision medicine studies. Our framework uses public genomic data sets of more than 11 000 tumor RNA-Seq samples that we consolidated and released to the community. We describe an application of our framework and the data compendium to the analysis of 144 tumors from children and young adults with a relapsed, refractory, or rare cancer, studied on 4 separate precision medicine trials in the United States and Canada.

While genomic profiling of tumors is becoming the standard of care in oncology, many tumors, especially in children, do not harbor actionable DNA aberrations. Tumor gene expression information may increase the number of actionable aberrations detected in tumors, and its utility is being evaluated in adults (eg, the WINTHER trial^1^). Results of several studies suggested the possible clinical utility of RNA-Seq for children. The Michigan Oncology Sequencing Center's Peds-MiOncoSeq study^2^ evaluated 92 patients with relapsed or refractory tumors using a combination of whole-exome sequencing (WES) and RNA-Seq and reported that 46% of samples had actionable findings, including 36% of this subset that had gene fusions with a known or suspected role in tumorigenesis identified through RNA-Seq analysis. In another study^3^ of 59 children, most with relapsed or refractory cancers, analysis revealed actionable findings, including RNA fusions, in 51% of cases. The Individualized Therapy for Relapsed Malignancies in Childhood (INFORM) consortium^4^ studied 57 patients with WES, low-coverage whole-genome sequencing, RNA-Seq, methylation, and gene expression microarrays and reported a 50% rate of actionable findings that included overexpression of druggable oncogenes. Several patients whose tumors exhibited oncogene overexpression were placed on targeted therapies against these alterations.^4^ Finally, the Precision in Pediatric Sequencing (PIPseq) program^5^ profiled 65 patients using a combination of tumor or normal WES and tumor RNA-Seq. Tumor RNA-Seq identified therapeutic targets in 23% of the patients; these targets included overexpression of druggable oncogenes, defined based on comparisons of tumor RNA-Seq expression with the RNA-Seq expression levels in a panel of normal tissues. While results of these studies suggested that RNA-Seq expression may be clinically beneficial, they did not provide reproducible methods that could be applied across different precision medicine trials.

Our group recently developed a reproducible and scalable approach for performing outlier analysis for pediatric patients with cancer by using large publicly available cancer RNA-Seq data sets.^6^ The objective of the present study was to evaluate the feasibility and potential utility of our approach for cancer samples collected prospectively from multiple precision medicine trials in difficult-to-treat pediatric and young adult patients with cancer.

## Methods

### Study Design

Among 144 tumors from children and young adults, this cohort study was conducted as a consortium of the following 4 clinical sites: British Columbia Children’s Hospital (BCCH), Vancouver, British Columbia, Canada; Lucile Packard Children’s Hospital at Stanford University (LPCH), Stanford, California; CHOC Children’s Hospital and Hyundai Cancer Institute, Orange, California; and the Pacific Pediatric Neuro-Oncology Consortium (PNOC), San Francisco, California. During the period from January 1, 2016, to March 22, 2017, the University of California, Santa Cruz (UCSC) obtained and processed tumor RNA-Seq data, as well as deidentified clinical and molecular information, for 181 tumors from 161 children and young adults with a relapsed, refractory, or rare cancer treated on precision medicine protocols. Tumor RNA-Seq data were obtained from the following 4 clinical sites: BCCH (n = 31), LPCH (n = 80), CHOC (n = 46), and PNOC (n = 24). Each clinical site had its own precision medicine protocol in place, and UCSC Treehouse Childhood Cancer Initiative served as a third-party institution conducting secondary analysis of each site’s tumor RNA-Seq data. This study followed the Strengthening the Reporting of Observational Studies in Epidemiology (STROBE) reporting guideline.

The BCCH study was approved by the University of British Columbia Research Ethics Committee. The LPCH protocol “Clinical Implementation of Genomic Analysis in Pediatric Malignancies” was approved by the Stanford University Institutional Review Board. The CHOC study “Pilot Project: Molecular Profiles of Newly Diagnosed, Refractory and Recurrent Childhood, Adolescent, and Young Adult Cancers” was approved by the CHOC Children’s Hospital and Hyundai Cancer Institute Institutional Review Board. The PNOC-003 protocol has been previously described.^7^ The UCSC Treehouse Childhood Cancer Initiative protocol was approved by the UCSC Institutional Review Board.

Because this study involved the sharing of deidentified data, UCSC was not required by our institutional review board to obtain informed consent from study participants; however, clinical partners obtained written informed consent from their participants as per their individual study protocols. All study participants were informed that their deidentified data would be shared with research partners, including UCSC.

### Statistical Analysis

#### Comparative RNA-Seq Analysis

All RNA-Seq data (11 340 compendium samples and 144 samples from clinical partners) were first uniformly processed using the RNA-Seq pipeline version 3.2 developed by the UCSC Computational Genomics Lab^8^ (eMethods in the Supplement). The UCSC either downloaded RNA-Seq data from a partner institution for analysis in the cloud or provided a Docker pipeline composed of gene-level expression calculation, which was run at the partner institution; gene expression outlier analysis and identification of druggable genes and pathways was then run on each of the 144 samples at UCSC.

#### Gene Expression Outlier Analysis

Gene-level transcript per million data were used to perform gene expression outlier analysis^9^ to identify transcripts significantly enriched in each patient’s tumor compared with either all 11 340 tumors or tumor types identified as most similar (pan-disease analysis). For pan-cancer analysis, we used the filtered set of 27 084 genes; for pan-disease analysis, we used the unfiltered set of 58 581 unique GENCODE Human Release 23 genes (eMethods in the Supplement) to make sure we did not miss genes whose expression is specific to certain tumor subtypes.

#### Identification of Druggable Overexpressed Genes and Gene Sets

We obtained the following 3 lists of overexpressed genes: one list from pan-disease outlier analysis, a second list from pan-cancer outlier analysis, and a third list from overlapping genes in pan-disease and pan-cancer lists. For each list, we identified potential druggable genes and statistically enriched pathways.

#### Drug-Gene Interaction Analysis

We used the Drug-Gene Interaction Database to assess which of the overexpressed genes can be considered actionable by available therapies.^10^ The database programmatically searches through publications and other curated databases for reported associations between human genes and available inhibitors. To refine our findings to only existing cancer therapies, we set the Drug-Gene Interaction Database to query for drug-gene interactions among the following 4 curated cancer databases (all part of the Drug-Gene Interaction Database^10^): CIViC, Cancer Commons, My Cancer Genome, and My Cancer Genome Clinical Trial. The Drug-Gene Interaction Database does not contain all known drug-gene interactions, nor does it guarantee a gene’s druggability. As a result, we performed additional literature searches and consulted published clinical cancer genomic studies. We prioritized studies, such as INFORM,^4^ in which gene expression information was considered in assessing the actionability of each gene. The 92 genes for which overexpression was considered directly or indirectly actionable in this study are listed in eTable 1 in the Supplement.

#### Gene Set Overlap Analysis

In parallel to identifying druggable genes, we used the Molecular Signature Database^11^ to identify overexpressed cancer pathways in the tumor sample. Gene set overlap analysis computes statistically significant pathways by evaluating the overlap between the input gene list of overexpressed genes and the gene sets from the Molecular Signature Database^11^ collections “Hallmark Gene Sets” and “Canonical Pathways.” In this analysis, for each input gene list, we looked at the first 100 reported gene sets that have the false discovery rate (false discovery rate *q* value) below 0.05.

#### DNA Mutation Analysis

DNA mutation data were obtained from the following platforms: Foundation Medicine gene panel (LPCH), whole-genome sequencing as part of the Personalized Onco-Genomics Program (POG) (BCCH), NantOmics whole-genome sequencing (CHOC), or Ashion Analytics whole-exome sequencing (PNOC). We used the National Cancer Institute (NCI) Pediatric Molecular Analysis for Therapeutic Choice (hereinafter the NCI Pediatric MATCH) considerations to curate the mutation data reported by the DNA platforms and to classify samples into treatment arms based on the DNA aberrations.^12^

## Results

### Patient Characteristics

**Figure 1.**
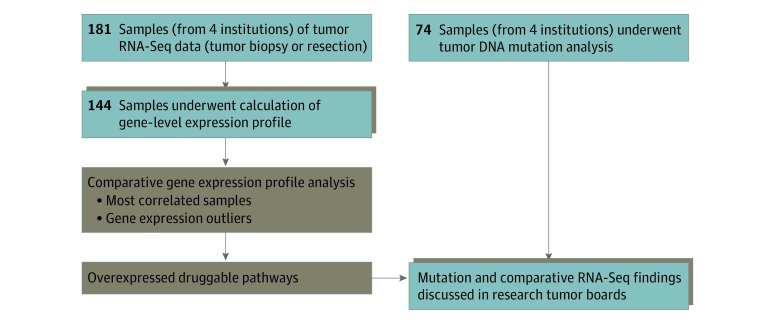
Treehouse Workflow The components in brown are performed by the University of California, Santa Cruz bioinformatics team, while the components in gray are performed by the clinical partners. Calculation of gene-level expression profiles can occur at the University of California, Santa Cruz or at a partner site through the use of portable software. Both the University of California, Santa Cruz and clinical partners participate in research discussions about cases. RNA-Seq indicates RNA sequencing.

To evaluate the feasibility of comparative RNA-Seq analysis across multiple precision medicine studies, we obtained RNA-Seq data from 181 samples from 161 pediatric and young adult patients (age range, 0-29 years; 65 of 108 [60.2%] male) with a relapsed, refractory, or rare cancer treated at the following 4 clinical sites: BCCH (n = 31), LPCH (n = 80), CHOC (n = 46), and PNOC (n = 24). The age at diagnosis was available for 126 individuals: the median age at diagnosis was 9 years, and the range was 0 to 26 years. Among 144 tumor samples, 46 were from female patients, while 72 were male patients; sex was not reported for 26 samples. RNA sequencing quality control analysis (eMethods in the Supplement) was applied to all 181 samples; of these, 144 samples from 128 patients were of sufficient quality for further analysis. For each case, gene-level transcript per million measurements were computed^8^ from tumor RNA-Seq data, which were used in 2 types of analyses to identify expression features of potential clinical relevance (Figure 1).

### Reference Compendium for Tumor Comparisons

To provide a robust reference for tumor comparisons and gene expression outlier detection, we assembled a compendium of 11 340 uniformly analyzed adult, pediatric, and young adult tumor profiles (eTable 2 and eFigure 1 in the Supplement). Of 11 340 samples in the compendium, 1859 (16.4%) were from pediatric, adolescent, and young adult patients with cancer who were younger than 30 years.

### Gene Expression Outlier Analysis

Gene expression outlier analysis is a promising method for identifying druggable overexpressed oncogenes in adult tumors.^9,13^ We performed gene expression outlier analysis against similar tumors (pan-disease analysis) and against all cancers in our compendium (pan-cancer analysis) (eMethods in the Supplement).

The gene expression outliers were analyzed for the presence of genes whose products could be targeted by small molecules directly or indirectly by targeting the downstream signaling pathway (eTable 1 in the Supplement). This list is based on a similar list prepared by the INFORM study^4^ and contains 37 genes whose protein products can be targeted directly and 55 genes whose products cannot be targeted but that function in a pathway that can be targeted by a therapy. We hypothesized that aberrant gene dosage of these directly or indirectly actionable genes could be detected by gene expression outlier analysis. We also sought to assess whether multiple members of the same pathways were highly expressed in concert in the same tumor.

**Figure 2.**
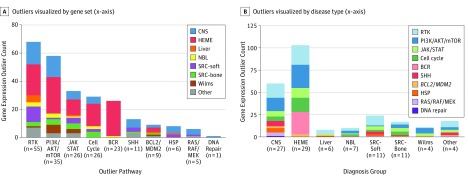
Actionable Gene Expression Outliers Identified Through Comparative RNA Sequencing Analysis of the Cohort The details of findings in each sample are listed in eTable 3 in the Supplement. BCR indicates B-cell receptor; CNS, central nervous system tumors; HEME, hematopoietic tumors; HSP, heat-shock proteins; JAK/STAT, Janus kinase and signal transducer and activator of transcription signaling pathway; NBL, neuroblastomas; PI3K/AKT/mTOR, phosphatidylinositol-3-kinase (PI3K)/AKT and the mammalian target of rapamycin (mTOR) signaling pathway; RAS/RAF/MEK, mitogen-activated protein kinase RAS/RAF/MEK/ERK pathway; RTK, receptor tyrosine kinases; SHH, sonic hedgehog; and SRC, sarcomas.

**Figure 3.**
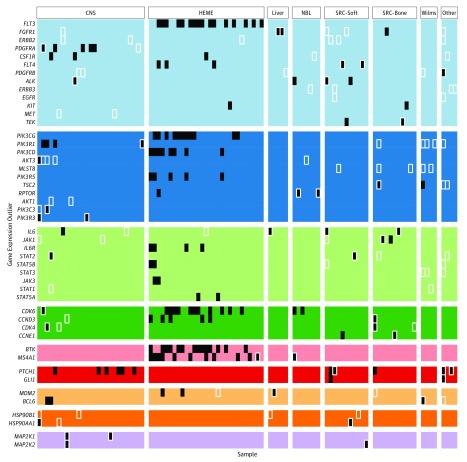
Recurrent Actionable Gene Expression Outliers Recurrent actionable gene expression outliers (y-axis), colored by gene sets as in Figure 2B, organized by disease (x-axis). Filled black squares denote outliers identified using the pan-cancer analysis approach, while unfilled white squares denote outliers identified by the pan-disease analysis approach. CNS indicates central nervous system tumors; HEME, hematopoietic tumors; NBL, neuroblastoma; and SRC, sarcoma.

Of 144 high-quality RNA-Seq data sets, 99 (68.8%) harbored outlier gene expression of 1 of 92 actionable genes. In 75 samples, both an actionable gene and the corresponding pathway were overexpressed using outlier analysis. The most common gene expression outlier was *FLT3* (OMIM 136351), overexpressed in 16 samples, all from hematopoietic tumors. This was followed by *BTK* (OMIM 300300) and *CDK6* (OMIM 603368), overexpressed in 14 samples each. While *BTK* was overexpressed in 14 hematopoietic tumors, *CDK6* was overexpressed in both hematopoietic and nonhematopoietic tumors, including neuroblastoma and glioma. The most common gene expression outlier in nonhematopoietic tumors was *PTCH1* (OMIM 601309), overexpressed in 11 samples from craniopharyngioma, neurofibroma, sarcoma, glioma, medulloblastoma, and osteosarcoma. The most common overrepresented gene set was receptor tyrosine kinases, overexpressed in 55 samples from all diagnostic categories (Figure 2). Among these, *FLT3* was most commonly overexpressed, followed by *FGFR1* (OMIM 136350) and *PDGFRA* (OMIM 173490). While *FGFR1* was overexpressed in a variety of nonhematopoietic tumor types, *PDGFRA* was exclusively overexpressed in brain tumors, and *FLT3* was exclusively overexpressed in acute leukemias. Of the 92 actionable genes, 47 were overexpressed in 2 or more samples (Figure 3). For the remaining 45 of the 144 samples (31.3%), our comparative RNA-Seq analysis did not identify any actionable outliers (eTable 3 in the Supplement). An example of Treehouse analysis is provided in eFigure 2 in the Supplement.

### Comparison of RNA-Seq Findings With DNA Mutation Analysis

A small number of childhood tumors contain DNA alterations that may forecast response to molecularly targeted therapies.^14^ Children’s Oncology Group NCI Pediatric MATCH^12^ is a nationwide basket trial for children and adolescents with relapsed or refractory solid tumors evaluating the use of DNA analysis to match patients to therapies. We had mutation data available for 74 of the 144 samples in our cohort; 52 of 74 were solid tumors.

**Figure 4.**
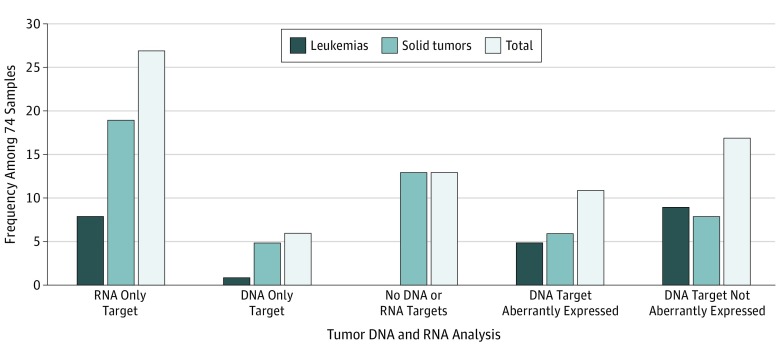
Comparison of DNA and RNA Analysis Results DNA and RNA analysis results were reviewed for 74 samples with both types of data available.

**Figure 5.**
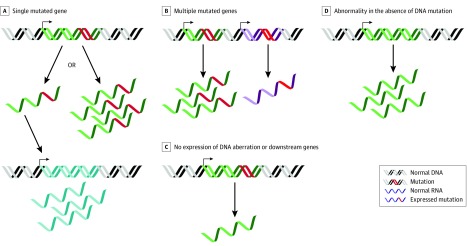
Utility of RNA Sequencing (RNA-Seq) Analysis A, RNA-Seq analysis can be used as additional support for DNA aberrations when a single mutated gene is itself highly expressed or downstream genes are highly expressed as a result of the mutation. B, With multiple mutated genes, RNA-Seq analysis can be used to prioritize among them based on high expression of the mutated gene itself or downstream targets. C, If DNA aberration is not expressed, nor are downstream genes, RNA-Seq analysis can be used to deprioritize DNA abnormalities with no evidence of effectiveness at the level of RNA. D, RNA-Seq analysis can reveal an abnormality in the absence of DNA mutation.

Of 74 solid tumor and leukemia samples, 34 (45.9%) had an actionable abnormality as defined by the NCI Pediatric MATCH study^12^ detected by DNA analysis. Fifty-five of 74 samples (74.3%) had an actionable gene expression outlier (eTable 3 in the Supplement) detected by RNA-Seq, 28 (37.8%) had abnormalities detected by both DNA and RNA analysis, 6 (8.1%) had only DNA abnormalities, and 13 (17.6%) had no DNA or RNA abnormalities. Remarkably, 27 samples (36.5%) had only a gene expression dosage abnormality, highlighting the potential utility of comparative RNA-Seq for nominating molecular targets for patients with no DNA findings (Figure 4 and Figure 5).

To assess the consistency of DNA and RNA findings, we reviewed 28 samples that had both types of findings. In 11 of 28 samples, at least 1 of the genes with a targetable DNA mutation was identified as a gene expression outlier, suggesting that actionable DNA mutations are often associated with the overexpression of the mutated gene. In 17 of 28 samples, however, none of the genes with a targetable DNA abnormality were identified as a gene expression outlier. Because we do not necessarily expect all mutant genes to be abnormally expressed themselves, we then reviewed the 17 samples to see if there was expression support of the DNA abnormality downstream of the mutated gene.

DNA analysis of 2 acute lymphoblastic leukemia samples (TH01_0122_S01 and TH01_0130_S01) revealed a *PAX5* (OMIM 167414)–*JAK2* (OMIM 147796) fusion, which was previously shown to activate Janus kinase and signal transducer and activator of transcription (JAK/STAT) signaling and promote a progenitor phenotype in leukemia cells.^15^ Our comparative gene expression analysis did not reveal the overexpression of the JAK/STAT pathway in these tumors but instead identified overexpression of phosphatidylinositol-3-kinase (PI3K)/AKT and the mammalian target of rapamycin (mTOR) (PI3K/AKT/mTOR) signaling pathway and B-cell receptor signaling pathways in both tumors and overexpression of *FLT3* in TH01_0130_S01. The overexpression of PI3K/AKT/mTOR and B-cell receptor signaling pathway genes may be indicative of a progenitor B-cell state assumed by the leukemia cells.^16^ Similarly, another acute lymphoblastic leukemia sample (TH01_0129_S01) harbored a *BCR*–*ABL* (OMIM 151410) fusion. RNA sequencing revealed outlier expression of PI3K/AKT/mTOR and B-cell receptor signaling pathways; PI3K/AKT/mTOR activation is known to be downstream of the *BCR*-*ABL* fusion signaling,^17^ suggesting that this overexpression is consistent with the DNA finding of the gene fusion. DNA analysis of 5 leukemia samples (TH01_0124_S01, TH01_0134_S01, TH03_0010_S01, TH03_0010_S02, and TH03_0011_S01) identified an activating mutation in *NRAS* (OMIM 164790). Activation of *NRAS* has been associated with proliferation and self-renewal in leukemia via the activation of MEK and mTOR signaling pathways.^18^ Our RNA-Seq analysis revealed overexpression of cell cycle or *BCL2* (OMIM 603167)–*MDM2* (OMIM 164785) pathways in TH01_0134_S01, TH03_0010_S01, TH03_0010_S02, and TH03_0011_S01; these pathways are downstream of activated RAS signaling, and their overexpression is thus consistent with the activating *NRAS* mutation. Notably, TH01_0124_S01 harbored subclonal activating mutations in both *KRAS* (OMIM 190070) and *NRAS* (20.6% and 29.1% mutant allele frequency based on RNA-Seq, respectively). While gene expression analysis revealed overexpression of *FLT3*, outlier expression associated with pathways downstream of activated RAS signaling was not found. These findings may represent either discordance between the DNA and RNA analysis or intratumor heterogeneity in this leukemia sample, already suspected based on the presence of 2 subclonal RAS mutations.

DNA analysis of a diffuse intrinsic pontine glioma (DIPG), TH02_0092_S01, revealed copy number gains of *KDR* (OMIM 191306), *KIT* (OMIM 164920), and *PDGFRA*, located on 4q12. Notably, while *KIT* and *PDGFRA* were highly expressed but not meeting the outlier threshold (84th and 93rd percentiles in the compendium), *KDR* was expressed at a much lower level, in the 54th percentile. Therefore, considering expression information alongside the copy number information may be useful for prioritizing druggable targets within copy number amplicons.^19^ In another DIPG sample, TH02_0091_S01 with a *BRAF* (OMIM 164757) p.V600E mutation, gene expression analysis revealed outlier expression of *CSF1R* (OMIM 164770). Recent work in melanoma showed that overexpression of *CSF1R* can occur in melanomas with activating *BRAF* or MAPK mutations and is associated with resistance to BRAF inhibitors.^20^ Because the interaction of these 2 pathways in DIPG is not known, we did not consider these concordant DNA and RNA findings.

An atypical teratoid rhabdoid tumor (TH03_0016_S01) and myoepithelial carcinoma (TH03_0113_S01) harbored loss of *SMARCB1* (OMIM 601607) (INI1) through a frameshift mutation or protein loss of unknown mechanism detected by immunohistochemistry, respectively. Comparative gene expression analysis of both tumors revealed outlier expression of *FGFR1*, a promising target in rhabdoid tumors deficient in *SMARCB1* (INI1).^21^ Gene expression analysis of DIPG tumor TH02_0087_S01 with a loss-of-function mutation of *PIK3R1* (OMIM 171833) activating the PI3K/AKT/mTOR pathway revealed overexpression of the JAK/STAT pathway. While it is unknown whether PI3K/AKT/mTOR and JAK/STAT pathways interact in DIPG, these pathways may be coactivated as a result of PI3K mutations in meningiomas.^22^ Because the interaction of these 2 pathways in DIPG is not known, we did not count this sample as having concordant DNA and RNA findings. Comparative gene expression analysis of a malignant peripheral nerve sheath tumor TH06_0645_S01 and neurofibroma TH06_0646_S01 with loss of *NF1* (OMIM 162200) revealed overexpression of sonic hedgehog signaling present in this tumor type.^23^ We also identified overexpression of receptor tyrosine kinases *ERBB3* (OMIM 190151) and *EGFR* (OMIM 131550) in these tumors.

Finally, in a glioma TH03_0290_S01 with a *BRAF* p.V600E mutation, the mutation was not expressed in the RNA. In an additional case (TH01_0131_S01), an activating *JAK2* mutation was supported by only a few reads, with more than 100 total read coverage in both the DNA and RNA, suggesting that the mutation may represent a subclonal event or a technical artifact.

Overall, our review of 17 samples with mutated genes not themselves overexpressed by RNA-Seq analysis revealed that in 12 of the 17 samples the overexpressed genes and pathways were consistent with the detected DNA mutations, even though the mutant genes themselves were not overexpressed. In the remaining 5 samples, outlier expression was not consistent with an activating mutation detected in the sample (including the lack of a *BRAF* p.V600E mutant allele in the RNA in TH03_0290_S01; ambiguous evidence in TH02_0087_S01, TH01_0124_S01, and TH02_0091_S01; and possible technical issues in TH01_0131_S01).

## Discussion

DNA sequencing is increasingly integrated in clinical trials to identify new molecular targets for children with incurable cancers. However, molecular targets are found for only a small number of patients, and the yield is much lower than that of similar adult cancer trials.^24^ Studies focusing on pediatric cancers have shown that the percentage of patients with potentially actionable findings increases to 40% to 50% when RNA-Seq data are considered alongside DNA mutation information.^4^ Herein, we described a framework for including RNA-Seq–derived gene expression information into precision medicine studies. Most notably, we show for the first time to date that such a framework can be used consistently across separate precision medicine clinical trials.

To our knowledge, our work represents the first report of a translational cancer genomic analysis in which prospective patient data are analyzed by a third-party computational group, with results returned to clinicians and researchers. We found that this comparative analysis is feasible and can produce new information of potential clinical relevance in 68.8% of samples. In 36.5% of samples (27 of 74), druggable overexpressed genes and pathways were identified based on RNA analysis alone and were not apparent in the tumor DNA analysis. Our work suggests that direct investigations of the clinical utility and effectiveness of tumor RNA-Seq–derived gene expression information will be valuable, and the next phase of our project will focus on defining the incremental benefit of this approach. The findings from our work also suggest that open sharing of cancer genomic data can benefit each pediatric and young adult patient with cancer so that every family’s struggle contributes to the advancement of clinical care for the families that follow.

### Clinical Implications

Although this study was not designed to assess clinical consequences, we noted associations of comparative RNA-Seq analysis findings and clinical features. For example, our analysis of a high-risk neuroblastoma sample revealed outlier expression of the *ALK* (OMIM 105590) kinase and *CDK6* kinase (eFigure 2 in the Supplement). The outlier expression of *CDK6*, as well as several other cell cycle genes, was consistent with a known DNA amplification of *CDK6* in this sample; however, the potential activation of *ALK* (OMIM 191175) was not evident before the RNA analysis. In another example, a 2-year-old boy with multifocal stage 4 hepatoblastoma metastatic to the lungs, was initially treated in the Childhood Liver Tumour Strategy Group of the International Society of Paediatric Oncology (SIOPEL-4) study,^25^ followed by surgery, 2 cycles of HEP0731 regimen T protocol, then salvage therapy with 3 cycles of vincristine, irinotecan, and temozolomide and 1 cycle of gemcitabine-oxaliplatin with bevacizumab. The patient had disease progression despite these therapies. Pathological analysis showed well and poorly differentiated hepatoblastoma with fetal and embryonal elements, and immunostaining showed retention of INI1 staining and diffuse nuclear and cytoplasmic β-catenin. Foundation Medicine testing revealed the p.G34V variant in CTNNB1, previously reported in hepatocellular carcinoma as an activating mutation.^26^ Comparative RNA-Seq analysis of the liver sample (TH03_0004_S04) uncovered gene expression similar to the proliferation subtype of hepatocellular carcinoma^27,28^ as well as outlier expression of HSP90B1, interleukin 6, and 4 other members of the JAK/STAT pathway. The overexpression of HSP90B was previously noted in hepatocellular carcinoma.^29^ The proliferative subtype of hepatocellular carcinoma is characterized by increased proliferation, high levels of serum α-fetoprotein (AFP), and chromosomal instability^27^; tumors with chromosomal instability are potentially sensitive to Aurora kinase inhibitors.^30^ Consistent with the similarity of the tumor to the proliferative subtype of hepatocellular carcinoma, the patient with the TH03_0004_S04 tumor had a response to the pan-kinase inhibitor pazopanib hydrochloride, with activity against Aurora kinase A.^31^ Based on the present study, after initiation of this treatment, the patient had a decline in his AFP levels from 14 036 to 1052 ng/mL at 7 weeks after initiation of the therapy (to convert AFP level to micrograms per liter, multiply by 1.0). At 10 weeks into this therapy, restaging studies showed progressive disease, and the patient was switched to therapy with ruxolitinib phosphate, without objective response by AFP levels or by imaging criteria.

### Limitations

Our study has some limitations. The heterogeneous nature of the patients analyzed in this study (all types of relapsed, refractory, and rare cancers) made drawing general statements difficult. The study was not designed to directly evaluate clinical utility of comparative RNA-Seq analysis, and clinical follow-up data on these patients were not readily available.

## Conclusions

Our experience suggests that it is feasible to include RNA-Seq–derived gene expression analysis in precision medicine studies and that this analysis can be harmonized across studies. We showed that RNA-Seq–derived gene expression was potentially useful for 68.8% of 144 samples compared with DNA mutation information, which was potentially useful for only 45.9% of 74 samples. Our study also highlights for the first time to date the potential clinical utility of harmonized publicly available genomic data sets. Open sharing and combined analysis of tumor RNA-Seq data from pediatric and young adult patients treated on separate clinical trials represent a feasible approach and can produce useful clinical and biological information for individual patients.

## Notes

Supplement. **eMethods.** Supplemental Methods **eTable 1.** Directly and Indirectly Actionable Genes Used to Prioritize Gene Expression Outlier Findings **eTable 2.** Published Repository Data Sets Included in the Treehouse Reference Compendium v5 **eTable 3.** Findings From Comparative RNA-Seq Analysis and Comparisons to Mutation Analysis **eFigure 1.** Treehouse Reference Compendium Used for Cross-Tumor Comparisons **eFigure 2.** Treehouse Analysis of Sample TH03_0288_S01 **eReferences.**
