# Supplementary material for: Comparative Tumor RNA Sequencing Analysis for Difficult-to-Treat Pediatric and Young Adult Patients With Cancer
Source: JAMA Netw Open. 2019 Oct 25;2(10):e1913968. doi: 10.1001/jamanetworkopen.2019.13968 (PMC6822083; doi:10.1001/jamanetworkopen.2019.13968)
Supplement: Supplement. — eMethods. Supplemental Methods eTable 1. Directly and Indirectly Actionable Genes Used to Prioritize Gene Expression Outlier Findings eTable 2. Published Repository Data Sets Included in the Treehouse Reference Compendium v5 eTable 3. Findings From Comparative RNA-Seq Analysis and Comparisons to Mutation Analysis eFigure 1. Treehouse Reference Compendium Used for Cross-Tumor Comparisons eFigure 2. Treehouse Analysis of Sample TH03_0288_S01 eReferences. [file jamanetwopen-2-e1913968-s001.pdf]

Supplementary Online Content

Vaske OM, Bjork I, Salama SR, et al. Comparative tumor RNA sequencing analysis for difficult-to-treat pediatric and young adult patients with cancer. *JAMA Netw Open*. 2019;2(10):e1913968. doi:10.1001/jamanetworkopen.2019.13968

eMethods. Supplemental Methods

eTable 1. Directly and Indirectly Actionable Genes Used to Prioritize Gene Expression Outlier Findings

eTable 2. Published Repository Data Sets Included in the Treehouse Reference Compendium v5

eTable 3. Findings From Comparative RNA-Seq Analysis and Comparisons to Mutation Analysis

eFigure 1. Treehouse Reference Compendium Used for Cross-Tumor Comparisons

eFigure 2. Treehouse Analysis of Sample TH03_0288_S01

eReferences.

This supplementary material has been provided by the authors to give readers additional information about their work.

eMethods. Supplemental Methods

Patient data transfer

We obtained raw RNA-Seq data from clinical partners using AWS via Amazon S3 and external hard drives. For BCCH, a Canadian institution, due to international regulations that limit the transfer of raw genomic information, we packaged our analysis pipelines into Docker containers (www.docker.com) to ensure consistent reproducible execution of our process, and to alleviate the need to transfer raw data files internationally. The BCCH bioinformaticians ran the Docker-ized analysis pipeline on their servers and shared the resulting gene-level expression information (gene-level TPM) across the border.

RNA sequencing analysis pipeline

The RNA sequencing (RNA-Seq) analysis pipelines can be obtained with the following docker command: docker pull quay.io/ucsc_cgl/rnaseq-cgl-pipeline:3.2.1-1. Gene expression quantification was performed as follows: Adapters were removed with CutAdapt v1.9. Reads were then aligned by STAR v 2.4.2a using indices generated from the human reference genome GRCh38 and the human gene models GENCODE 23. RSEM Version 1.2.25 was used to quantify gene expression. Reference data sources: GRCh38 was downloaded from ftp://ftp.ncbi.nlm.nih.gov/genomes/archive/old_genbank/Eukaryotes/vertebrates_mammals/Homo_sapiens/GRCh38/seqs_for_alignment_pipelines/ GCA_000001405.15_GRCh38_no_alt_analysis_set.fna.gz on July 22, 2016. GENCODE 23 was downloaded from ftp://ftp.sanger.ac.uk/pub/gencode/Gencode_human/release_23/gencode.v23.annotation.gtf.gz July 22, 2016. The source code of the pipeline is available at https://github.com/UCSC-Treehouse/pipelines.

For quality assessment, the genome-aligned reads that are output by our RNA-Seq pipeline[1] were sorted by name using sambamba v0.6.1, piped to Samblaster 0.1.22 for duplicate marking and then sorted by coordinate by sambamba v0.6.1. The tools geneBody_coverage.py and read_distribution.py from RSeQC v2.7.10 were run on all BAM files.

A quality metric, the number of mapped, exonic, non-duplicate (MEND) reads, was estimated from read_distribution.py output using the script parseReadDist.R to do the following 1) Calculate the number of reads per tag (from the top table, "Total Reads"/"Total Tags") 2) Calculate the number of exonic tags (from the bottom table, the sum of the Tag_count column for the rows "CDS_Exons", "5'UTR_Exons" and "3'UTR_Exons"). 3) Estimate the number of MEND reads by multiplying and halving the two previously calculated numbers: "number of exonic tags" * "number of reads per tag" /2. The QC process is available as a stand-alone docker image at https://hub.docker.com/r/hbeale/treehouse_bam_qc/. The source code is at https://github.com/UCSC-Treehouse/bam_qc.

Datasets used for comparative RNA-Seq analysis

The RNA-Seq sample data with associated clinical metadata were managed using REDCap electronic data capture tools hosted at UCSC[2]. Version 5 of the Treehouse Gene Expression Reference Compendium, used in this manuscript, comprises 11,340 samples derived from TCGA (n=9806), TARGET (n=784), 17 additional public repository datasets (n=586) and 164 from our partner clinical sites (eTable 2). The gene expression compendium is publicly available through the UCSC Xena Browser at https://treehousegenomics.ucsc.edu/p/vaske-2019-comparative-tumor-RNA.

Visualization of the reference compendium using TumorMap

To aid in the molecular classification of tumors, we used the previously published TumorMap method[3]. Briefly, we first computed pairwise Spearman correlations between RNA-Seq-derived gene expression profiles of the tumors in our reference cohort (n = 11,340). This produced a square correlation matrix with 11,340 columns and 11,340 rows. We initially measured the expression of 60,498 GENCODE 23 genes (58,581 of these had uique identifiers) and then filtered out 31,497 genes with low expression or variance from the original 60,498 for a final count of 27,084 genes retained for analysis. The correlation matrix is used as input for the TumorMap, which is available at https://tumormap.ucsc.edu/?p=vaske-2019-comparative-tumor-RNA.

Defining similar tumors for pan-disease outlier analysis

To identify compendium tumor samples most similar to each index sample, TPM values from each of the 144 samples were used to compute Spearman correlations between the index sample and all the tumors in our compendium. We used the correlations of the index sample to the compendium to identify samples most similar to the index case. For each case we defined the most similar tumors in four ways: (1) all samples with the same histopathological classification as the index sample ("diagnosed disease"), (2) all

© 2019 Vaske OM et al. *JAMA Network Open*.

samples with a disease shared with the top six most correlated samples (MCS) ("inferred disease"), (3) all MCS with a correlation to the index sample in the top 5th percentile of all correlations in the compendium (first degree MCS) ("high stringency correlation") and (4) all samples that are first degree MCS to the first degree MCS of the index samples (first and second degree MCS) ("low stringency correlation"). One hundred and thirty six samples (out of 144) shared a diagnosed disease with another sample in the compendium, and, as expected, for 129 of those 136 samples, the stringent correlation-based cohort included at least one sample with the diagnosed disease. In 119 of the 136, the stringent correlation-based cohort also included samples with other diseases, suggesting that comparative gene expression analysis can be useful for identifying tumors with similar expression signatures regardless of diagnoses. The cohorts of similar samples were used in the gene expression outlier analysis described below.

© 2019 Vaske OM et al. *JAMA Network Open*.

eTable 1. Directly and indirectly actionable genes used to prioritize gene expression outlier findings.

Gene	Category	Druggability	Available approved therapy
JAK1	JAK/STAT	directly druggable gene	ruxolitinib, tofacitinib
JAK2	JAK/STAT	directly druggable gene	ruxolitinib, tofacitinib
JAK3	JAK/STAT	directly druggable gene	tofacitinib
STAT1	JAK/STAT	indirectly actionable gene (JAK)	
STAT2	JAK/STAT	indirectly actionable gene (JAK)	
STAT3	JAK/STAT	directly druggable gene	
STAT5A	JAK/STAT	indirectly actionable gene (JAK)	
STAT5B	JAK/STAT	indirectly actionable gene (JAK)	
IL6	JAK/STAT	directly druggable gene	siltuximab
IL6R	JAK/STAT	directly druggable gene	tocilizumab
EGFR	Receptor Tyrosine Kinase (RTK)	directly druggable gene	cetuximab, erlotinib, afatinib, lapatinib
ERBB2	Receptor Tyrosine Kinase (RTK)	directly druggable gene	afatinib, lapatinib
ERBB3	Receptor Tyrosine Kinase (RTK)	directly druggable gene	lapatinib
ALK	Receptor Tyrosine Kinase (RTK)	directly druggable gene	crizotinib, ceritinib
MET	Receptor Tyrosine Kinase (RTK)	directly druggable gene	cabozantinib, crizotinib
ROS1	Receptor Tyrosine Kinase (RTK)	directly druggable gene	cabozantinib, crizotinib, ceritinib
FLT1	Receptor Tyrosine Kinase (RTK)	directly druggable gene	pazopanib, regorafenib
FLT3	Receptor Tyrosine Kinase (RTK)	directly druggable gene	ponatinib, sorafenib, sunitinib
KDR	Receptor Tyrosine Kinase (RTK)	directly druggable gene	sorafenib, sunitinib
FLT4	Receptor Tyrosine Kinase (RTK)	directly druggable gene	cabozantinib, axitinib
PDGFRA	Receptor Tyrosine Kinase (RTK)	directly druggable gene	dasatinib, pazopanib, sorafenib
PDGFRB	Receptor Tyrosine Kinase (RTK)	directly druggable gene	dasatinib, pazopanib, sorafenib, sunitinib
FGFR1	Receptor Tyrosine Kinase (RTK)	directly druggable gene	pazopanib, panopanib, regorafenib
KIT	Receptor Tyrosine Kinase (RTK)	directly druggable gene	imatinib, sunitinib, regorafenib
CSF1R	Receptor Tyrosine Kinase (RTK)	directly druggable gene	pazopanib
TEK	Receptor Tyrosine Kinase (RTK)	directly druggable gene	
CDK4	Cell Cycle	directly druggable gene	palbociclib, ribociclib
CDK6	Cell Cycle	directly druggable gene	palbociclib, ribociclib
CCND1	Cell Cycle	indirectly actionable gene (CDK)	
CCND2	Cell Cycle	indirectly actionable gene (CDK)	
CCND3	Cell Cycle	indirectly actionable gene (CDK)	
CCNE1	Cell Cycle	indirectly actionable gene (CDK)	
CDK2	Cell Cycle	directly druggable gene	
PIK3CA	PI3K/AKT/mTOR	directly druggable gene	idelalisib
PIK3CB	PI3K/AKT/mTOR	directly druggable gene	idelalisib

© 2019 Vaske OM et al. *JAMA Network Open*.

Gene	Category	Druggability	Available approved therapy
PIK3CG	PI3K/AKT/mTOR	directly druggable gene	idelalisib
PIK3CD	PI3K/AKT/mTOR	directly druggable gene	idelalisib
PIK3R1	PI3K/AKT/mTOR	indirectly actionable gene (mTOR)	
PIK3R2	PI3K/AKT/mTOR	indirectly actionable gene (mTOR)	
PIK3R3	PI3K/AKT/mTOR	indirectly actionable gene (mTOR)	
PIK3R4	PI3K/AKT/mTOR	indirectly actionable gene (mTOR)	
PIK3R5	PI3K/AKT/mTOR	indirectly actionable gene (mTOR)	
PIK3R6	PI3K/AKT/mTOR	indirectly actionable gene (mTOR)	
PIK3C2A	PI3K/AKT/mTOR	indirectly actionable gene (mTOR)	
PIK3C2B	PI3K/AKT/mTOR	indirectly actionable gene (mTOR)	
PIK3C2G	PI3K/AKT/mTOR	indirectly actionable gene (mTOR)	
PIK3C3	PI3K/AKT/mTOR	indirectly actionable gene (mTOR)	
AKT1	PI3K/AKT/mTOR	indirectly actionable gene (mTOR)	
AKT3	PI3K/AKT/mTOR	indirectly actionable gene (mTOR)	
TSC1	PI3K/AKT/mTOR	indirectly actionable gene (mTOR)	
TSC2	PI3K/AKT/mTOR	indirectly actionable gene (mTOR)	
MTOR	PI3K/AKT/mTOR	directly druggable gene	everolimus, temsirolimus
RPTOR	PI3K/AKT/mTOR	indirectly actionable gene (mTOR)	
MLST8	PI3K/AKT/mTOR	indirectly actionable gene (mTOR)	
DEPTOR	PI3K/AKT/mTOR	indirectly actionable gene (mTOR)	
NRAS	RAS/RAF/MEK	indirectly actionable gene (MEK)	
KRAS	RAS/RAF/MEK	indirectly actionable gene (MEK)	
HRAS	RAS/RAF/MEK	indirectly actionable gene (MEK)	
BRAF	RAS/RAF/MEK	directly druggable gene	dabrafenib
RAF1	RAS/RAF/MEK	directly druggable gene	dabrafenib, sorafenib, regorafenib
ARAF	RAS/RAF/MEK	indirectly actionable gene (MEK)	
MAP2K1	RAS/RAF/MEK	directly druggable gene	trametinib
MAP2K2	RAS/RAF/MEK	directly druggable gene	trametinib
PTCH1	Sonic Hedgehog (SHH)	indirectly actionable gene (SMO)	
GLI1	Sonic Hedgehog (SHH)	indirectly actionable gene (SMO)	
SMO	Sonic Hedgehog (SHH)	directly druggable gene	vismodegib
BCL2	BCL2/MDM2	directly druggable gene	venetoclax
BCL6	BCL2/MDM2	indirectly actionable gene (BCL2)	
MDM2	BCL2/MDM2	indirectly actionable gene (BCL2)	
HSP90AA1	Heat Shock Proteins	directly druggable gene	
HSP90AA2P	Heat Shock Proteins	directly druggable gene/gene family	
HSP90N	Heat Shock Proteins	directly druggable gene/gene family	
HSP90AA2	Heat Shock Proteins	directly druggable gene/gene family	

© 2019 Vaske OM et al. *JAMA Network Open*.

Gene	Category	Druggability	Available approved therapy
HSP90AA6P	Heat Shock Proteins	directly druggable gene/gene family	
HSP90AA3P	Heat Shock Proteins	directly druggable gene/gene family	
HSP90AA4P	Heat Shock Proteins	directly druggable gene/gene family	
HSP90AA5P	Heat Shock Proteins	directly druggable gene/gene family	
HSP90AB1	Heat Shock Proteins	directly druggable gene/gene family	
HSP90AB3P	Heat Shock Proteins	directly druggable gene/gene family	
HSP90AB2P	Heat Shock Proteins	directly druggable gene/gene family	
HSP90AB6P	Heat Shock Proteins	directly druggable gene/gene family	
HSP90AB4P	Heat Shock Proteins	directly druggable gene/gene family	
HSP90AB5P	Heat Shock Proteins	directly druggable gene/gene family	
HSP90B1	Heat Shock Proteins	directly druggable gene/gene family	
HSP90B2P	Heat Shock Proteins	directly druggable gene/gene family	
HSP90B3P	Heat Shock Proteins	directly druggable gene/gene family	
BTK	B-cell receptor signaling	directly druggable gene/gene family	ibrutinib
MS4A1	B-cell receptor signaling	directly druggable gene/gene family	rituximab
AURKA	Aurora kinases	directly druggable gene/gene family	
AURKB	Aurora kinases	directly druggable gene/gene family	
AURKC	Aurora kinases	directly druggable gene/gene family	
PARP1	DNA repair	directly druggable gene/gene family	olaparib

© 2019 Vaske OM et al. *JAMA Network Open*.

eTable 2. Published repository datasets included in the Treehouse Compendium v5.

A. Compendium v5 Repository Data

TH dataset	Accession	Repository	Project	Disease	N
TARGET	TARGET	dbGap	TARGET	multiple cancers	784
TCGA	TCGA	dbGap	TCGA	multiple cancers	9,806
THR08	EGAD00001001098	EGA	St Jude PCGP	ALL with MLL rearrangements	63
THR09	EGAD00001000356	EGA	ICGC	B-cell lymphoma	23
THR10	EGAD00001002680	EGA	St Jude PCGP	adrenocortical carcinoma	15
THR11	EGAD00001001666	EGA	St Jude PCGP	low grade glioma	32
THR13	phs000900.v1.p1	dbGap	PBTC (Monje Lab)	DIPG	10
THR14	CBTTC_HGG_PA_01	Cavatica	CBTTC	high grade glioma	29
THR15	phs000699.v1.p1	dbGap	Perry et al	osteosarcoma	29
THR17	EGAD00001000158	EGA	ICGC/MAGIC	medulloblastoma	17
THR18	EGAD00001000328	EGA	ICGC	medulloblastoma	6
THR19	EGAD00001000617	EGA	ICGC	pilocytic astrocytoma	69
THR20	EGAD00001001620	EGA	ICGC	medulloblastoma	39
THR22	EGAD00001000648	EGA	ICGC	germinal B-cell lymphoma	24
THR25	EGAD00001000826	EGA	ICGC	osteosarcoma	7
THR28	SRP040454	SRA	Kohsaka et al	rhabdomyosarcoma	3
THR29	phs000720.v2.p1	dbGap	Shern et al	rhabdomyosarcoma	83
THR30	phs000768.v2.p1	dbGap	Brohl et al	Ewing sarcoma	62
THR31	phs000673.v2.p1	dbGap	Peds Mi-OncoSeq/Met500	multiple cancers	75
				total	11,176
				total - (TCGA+TARGET)	586

B. Compendium v5 Treehouse Partner Data

TH dataset	Treehouse Partner	N
TH02	Pacific Pediatric Neuro-Oncology Consortium (PNOC)	29
TH03	Stanford, Sweet-Cordero Lab	73
TH04	Memorial Sloan Kettering Cancer Center via Stanford, Sweet-Cordero Lab	1
TH06	British Columbia Personalized Onco-Genomics (POG) Program	31
TH26	Stanford, Merker Lab	6
TH27	UC San Francisco, Sweet-Cordero Lab	24
	total	164

© 2019 Vaske OM et al. *JAMA Network Open.*

C. Compendium v5 Repository Data By Consortium

Consortium	v5 (Feb 2018)
TCGA	9,806
TARGET	784
ICGC	168
ICGC/MAGIC	17
St Jude PCGP	110
CBTTC	29
PBTC	10
other	252
total	11,176

D. Repository Data Acknowledgments

Treehouse expression dataset (v5, February 2018)

Use of data available in public repositories allowed us to increase the number of pediatric cancer patients and pediatric cancer types included in our reference compendium, and we gratefully acknowledge the following data providers in the manner they have specified:

"International Cancer Genome Consortium. The following ICGC datasets were used in this work: EGAD00001000158, EGAD00001001620, EGAD00001000328, EGAD00001000648, EGAD00001000617, EGAD00001000826 and EGAD00001000356."

"St. Jude Children's Research Hospital – Washington University Pediatric Cancer Genome Project. The following datasets were accessed via EGA (https://www.ebi.ac.uk/ega/) and were used by permission:

EGAD00001001098: Andersson AK, Ma J, Wang J, et al. The landscape of somatic mutations in infant MLL-rearranged acute lymphoblastic leukemias. Nat Genet. 2015;47(4):330-7. (PMC4553269).
EGAD00001001666: Qaddoumi I, Orisme W, Wen J, et al. Genetic alterations in uncommon low-grade neuroepithelial tumors: BRAF, FGFR1, and MYB mutations occur at high frequency and align with morphology. Acta Neuropathol. 2016;131(6):833-45. (PMID 26810070).
EGAD00001002680: Pinto EM, Chen X, Easton J, et al. Genomic landscape of paediatric adrenocortical tumours. Nat Commun. 2015;6:6302. (PMC4352712)."

"Provider(s) of the data under accession phs000178.v10.p8 at dbGap. The Cancer Genome Atlas Research Network
National Cancer Institute and National Human Genome Research Institute, Bethesda, MD, USA
The results published here are in whole or part based upon data generated by The Cancer Genome Atlas managed by the NCI and NHGRI. Information about TCGA can be found at http://cancergenome.nih.gov/."

"Provider(s) of the data under accession phs000178.v10.p8 at dbGap: The Therapeutically Applicable Research to Generate Effective Treatments (TARGET) initiative managed by the NCI. The data used for this analysis are available at dbGap under accession phs000218. Information about TARGET can be found at http://ocg.cancer.gov/programs/target.

© 2019 Vaske OM et al. *JAMA Network Open*.

Children's Oncology Group (COG; Tissues for TARGET are collected as part of COG clinical and biological protocols)
Peter C. Adamson, MD. Children's Hospital of Philadelphia, Philadelphia, PA, USA
Principal Investigator (ALL Project Team)
Stephen P. Hunger, MD. University of Colorado Cancer Center, Denver, CO, USA
Principal Investigators (AML Project Team)
Soheil Meshinchi, MD, PhD. Fred Hutchinson Cancer Research Center, Seattle, WA, USA
Robert Arceci, MD, PhD. Children's Hospital, Phoenix, AZ, USA
Principal Investigator (NBL Project Team)
John M. Maris, MD. Children's Hospital of Philadelphia, Philadelphia, PA, USA
Robert Seeger, MD. Children's Hospital of Los Angeles, Los Angeles, CA, USA
Javed Khan, MD. National Cancer Institute, National Institutes of Health, Bethesda, MD, USA
Principal Investigator (OS Project Team)
Ching Lau, MD, PhD. Texas Children's Hospital, Houston, TX, USA
Paul Meltzer, MD, PhD. National Cancer Institute, National Institutes of Health, Bethesda, MD, USA
Principal Investigator (Kidney Project Teams - WT, CCSK, RT)
Elizabeth J. Perlman, MD. Ann and Robert H. Lurie Children's Hospital of Chicago, Chicago, IL, USA
Principal Investigator (Cell Lines and Xenografts - PPTP)
Peter Houghton, PhD. The Research Institute at Nationwide Children's Hospital, Columbus, OH, USA"

Provider(s) of the data under accession phs000699.v1.p1 at dbGap: Todd Golub. Dana Farber Cancer Institute, Boston, MA, USA. This work was conducted as part of the Slim Initiative for Genomic Medicine in the Americas (SIGMA), a project funded by the Carlos Slim Health Institute in Mexico.

Provider(s) of the data under accession phs000900.v1.p1 at dbGap: Charles Keller. Children's Cancer Therapy Development Institute, Fort Collins, CO, USA. We thank the many patients and families who selflessly contributed to this study through tissue donations from surgery or autopsy and Amar Gajjar, for his guidance and vision throughout this study. We also thank Darren Hargrave, James Olson and Sarah Leary for selection of V.2 chemical screen agents. We are grateful for the critical questions and comments by Simone Cheetham and Nadim Nsouli. We also acknowledge important comments by other DIPG Preclinical Consortium member Oren Becher. We thank Gerald Grant for assistance in developing rodent CED techniques. Short read sequencing was performed by the OHSU Massively Parallel Sequencing Shared Resource. The first paper using this data was: Grasso CS, Tang Y, Truffaux N, et al. Functionally defined therapeutic targets in diffuse intrinsic pontine glioma. Nat Med. 2015;21(6):555-9. (PMID 25939062).

Provider(s) of the data under accession phs000720.v2.p1 at dbGap: Javed Khan, MD. National Institutes of Health, Bethesda, MD, USA. The authors thank the Children's Oncology Group Soft Tissue Sarcoma Committee and the BioPathology Center, for their careful collection of clinical samples. This research was supported by the Intramural Research Program of the National Institute of Health and National Cancer Institute.

Provider(s) of the data under accession phs000768.v2.p1 at dbGap. Javed Khan, MD. National Institutes of Health, Bethesda, MD, USA. This research was supported by the Intramural Research Program of the National Institute of Health and National Cancer Institute. The datasets have been accessed through the NIH database for Genotypes and Phenotypes (dbGaP) under accession # phs000768.v1.p1.

Provider(s) of the data under accession phs000673.v2.p1 at dbGap. Arul Chinnaiyan, MD PhD. Michigan Center for Translational Pathology, University of Michigan, MI, USA. The results published here are in whole or part based upon data generated by the Clinical Sequencing Exploratory Research (CSER) consortium

© 2019 Vaske OM et al. *JAMA Network Open*.

established by the NHGRI. Funding support was provided through cooperative agreements with the NHGRI and NCI through grant numbers U01 HG006508 (Exploring Cancer Medicine for Sarcoma and Rare Cancers). Information about CSER and the investigators and institutions who comprise the CSER consortium can be found at http://www.genome.gov/27546194

Provider(s) of the data under accession SRP040454 at SRA. Dataset was made available by Memorial Sloan Kettering Cancer Center.

This research was conducted using data made available by The Children's Brain Tumor Tissue Consortium (CBTTC).

a.

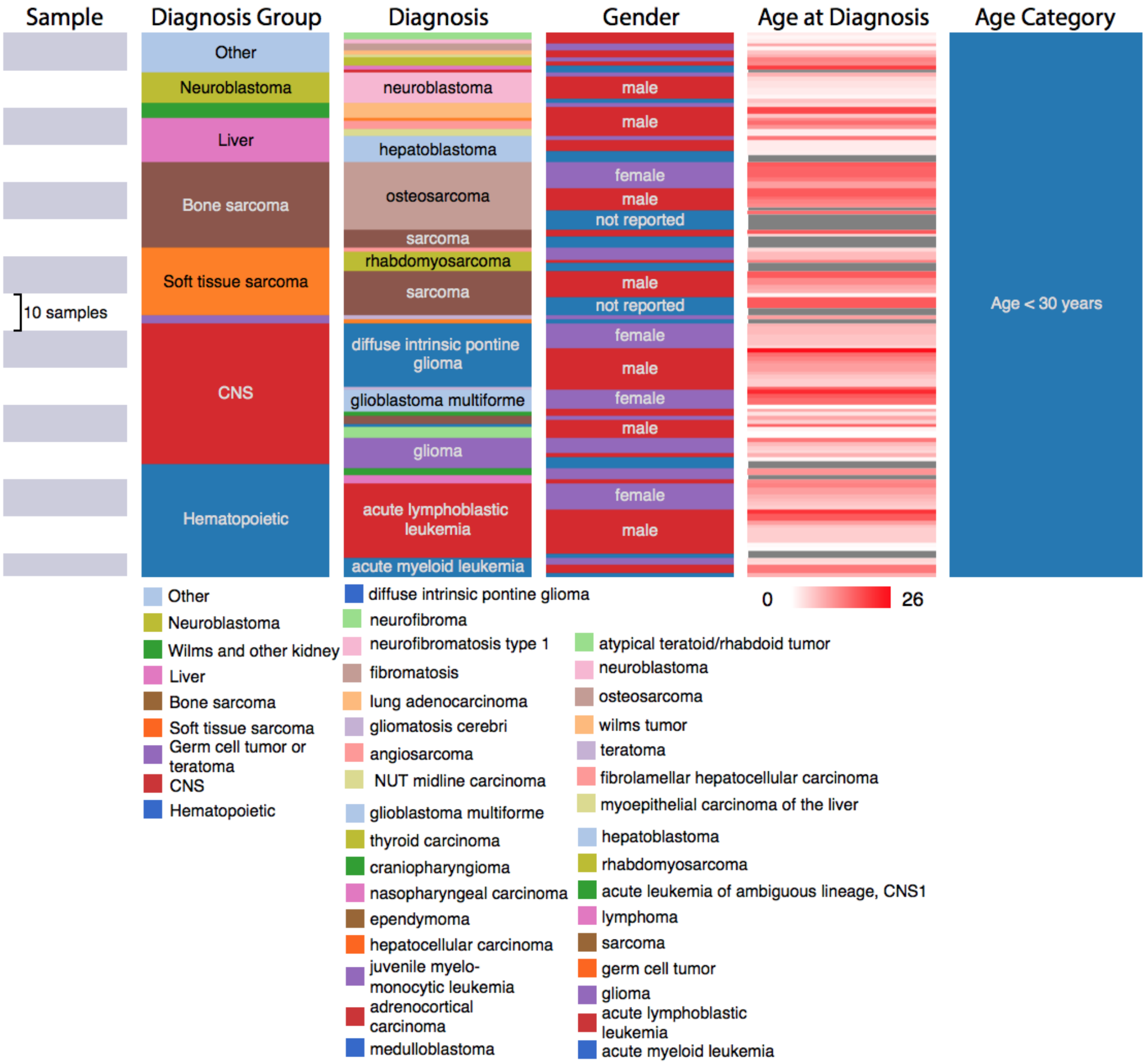

© 2019 Vaske OM et al. *JAMA Network Open*.

b.

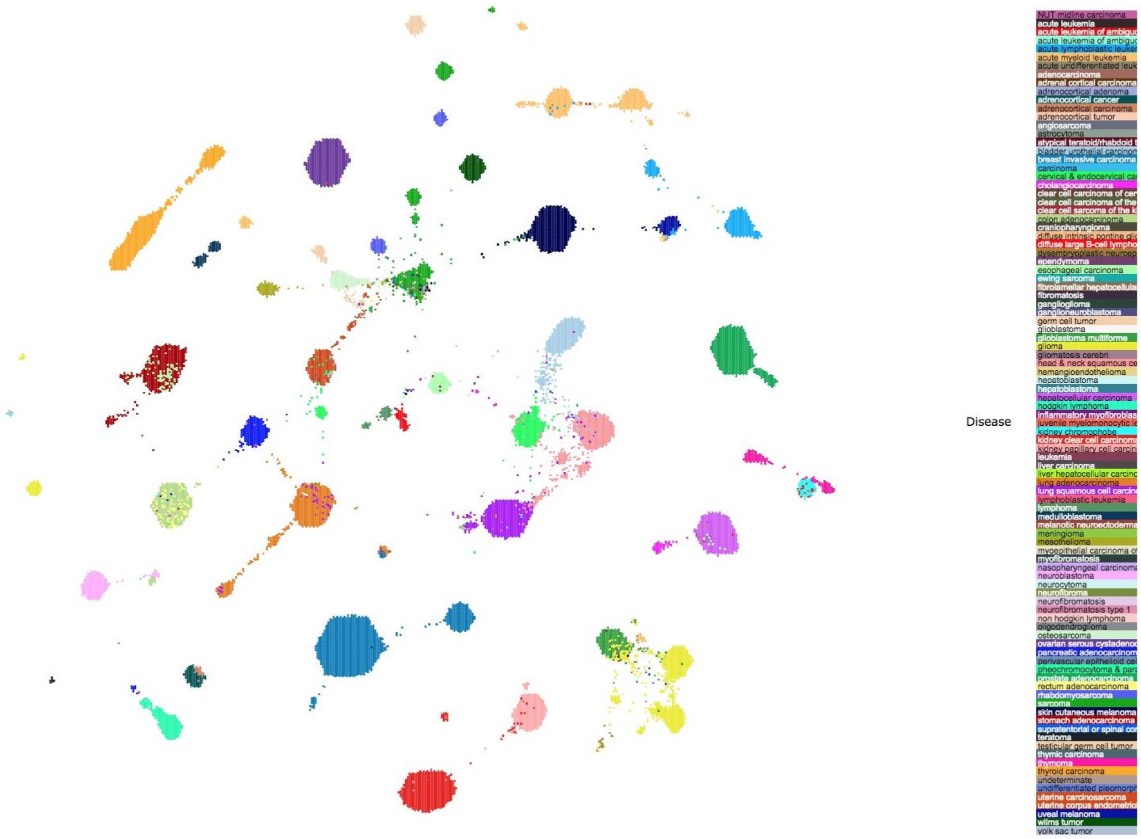

eFigure 1. Treehouse reference compendium used for cross-tumor comparisons. The data and visualizations are publicly available at treehousegenomics.ucsc.edu/p/vaske-2019-comparative-tumor-RNA. A. Treehouse prospective patient cohort (N=144) visualized using the UCSC Xena Browser (xena.ucsc.edu) B. Treehouse compendium (N=11,340) visualized using the TumorMap (eMethods). Each hexagon represents an individual sample and the samples are colored by disease as indicated in the legend.

© 2019 Vaske OM et al. *JAMA Network Open*.

eTable 3. Findings from comparative RNA-Seq analysis and comparisons to mutation analysis.

Panel A, "DNA-RNA comparison", details comparisons of DNA and RNA findings for 74 samples with both types of data.
Panel B contains results from comparative RNA-Seq analysis for all 144 samples.

A. DNA-RNA comparison for 74 samples with both types of data.

TH sample ID	Diagnosis	At least one DNA target aberrantly expressed?	Utility of RNA sequencing	Solid / leukemia	DNA mutation call of clinical relevance	DNA mutation category NCI Pediatric MATCH	Treehouse category	Outlier confirmed by pathway	Outlier not confirmed by pathway	gene	mutation	note	testVendor
TH01_0123_S01	acute lymphoblastic leukemia	Yes	Prioritizing among multiple DNA findings	leukemia						new sample			
					FLT3 amplification	Receptor Tyrosine Kinase (RTK)	Receptor Tyrosine Kinase (RTK)	FLT3, FLT4		FLT3	Low-levelAmplification		NantOmics
					JAK2 p.R683S	JAK/STAT	JAK/STAT	JAK3		JAK2	p.R683S		NantOmics
							B-cell receptor signaling	MS4A1		JAK2	p.R683S		NantOmics
							PI3K/AKT/mTOR	PIK3CD, PIK3CG, RPTOR					
TH01_0132_S01	acute myeloid leukemia	RNA only target	RNA only target	leukemia						new sample			
					No druggable mutations		B-cell receptor signaling	BTK		MLLT10-KMT2A	MLL rearrangement		NantOmics
							Cell cycle	CDK6					
							PI3K/AKT/mTOR	PIK3CG					
TH01_0129_S01	acute lymphoblastic leukemia	No	Additional expression support for DNA finding	leukemia						new sample			
					BCR-ABL1 fusion	Other							
							B-cell receptor signaling	MS4A1		BCR-ABL1			NantOmics
							PI3K/AKT/mTOR	PIK3CG					
TH01_0120_S01	acute myeloid leukemia	RNA only target	RNA only target	leukemia						new sample			
					No druggable mutations		Cell cycle	CDK6		GATA2	p.L321F		NantOmics
							Receptor Tyrosine Kinase (RTK)	KIT					
TH01_0121_S01	acute lymphoblastic leukemia	RNA only target	RNA only target	leukemia						new sample			
					No druggable mutations		JAK/STAT	IL6R, JAK3, STAT5B					NantOmics
							B-cell receptor signaling	MS4A1					
							PI3K/AKT/mTOR	PIK3CD, PIK3CG, PIK3R5					
TH01_0122_S01	acute lymphoblastic leukemia	No	Additional expression support for DNA finding	leukemia						new sample			
					PAX5-JAK2 fusion	JAK/STAT				PAX5-JAK2			NantOmics
							B-cell receptor signaling	MS4A1					
							PI3K/AKT/mTOR	PIK3CD, PIK3CG					
TH01_0124_S01	acute lymphoblastic leukemia	No		leukemia						new sample			
					KRAS p.G13D, NRAS p.G12D	RAS/RAF/MEK				KRAS	p.G13D		NantOmics
							Receptor Tyrosine Kinase (RTK)	FLT3		NRAS	p.G12D		NantOmics
TH01_0125_S01	acute lymphoblastic leukemia	RNA only target	RNA only target	leukemia						new sample			
					No druggable mutations		Receptor Tyrosine Kinase (RTK)	FLT3		CSF3R	p.D320N		NantOmics
										KMT2A-AFF1			NantOmics
							PI3K/AKT/mTOR	PIK3CG		TSC2	p. R367Q	Benign	NantOmics
							Cell cycle	CDK6					
							B-cell receptor signaling	BTK					
TH01_0126_S01	acute lymphoblastic leukemia	DNA only target	DNA only target	leukemia						new sample			
					NRAS p.G12D	RAS/RAF/MEK	No automated leads			NRAS	p.G12D		NantOmics
					CDKN2A deletion	Cell Cycle				CDKN2A	deletion		NantOmics
TH01_0128_S01	acute lymphoblastic leukemia	RNA only target	RNA only target	leukemia						new sample			
					No druggable mutations		JAK/STAT	IL6R					NantOmics
							PI3K/AKT/mTOR	PIK3CD, PIK3CG, PIK3R5					
TH01_0134_S01	acute lymphoblastic leukemia	No	Additional expression support for DNA finding	leukemia						new sample			
					NRAS p.G13D	RAS/RAF/MEK				NRAS	p. G13D		NantOmics
							Cell cycle	CDK6					
TH01_0133_S01	acute lymphoblastic leukemia	RNA only target	RNA only target	leukemia						new sample			
					No druggable mutations		Receptor Tyrosine Kinase (RTK)		FLT3				NantOmics
TH01_0131_S01	acute lymphoblastic leukemia	No	DNA finding likely a technical artifact	leukemia						new sample			

© 2019 Vaske OM et al. *JAMA Network Open*.

					JAK2 p.R683S	JAK/STAT				JAK2	p.R683S		NantOmics
							B-cell receptor signaling	BTK					
							Cell cycle	CDK6					
							Receptor Tyrosine Kinase (RTK)	FLT3, FLT4					
							PI3K/AKT/mTOR	PIK3CG					
TH01_0127_S01	acute lymphoblastic leukemia	Yes	Prioritizing among multiple DNA findings	leukemia						new sample			
					FLT3 p.V592D	Receptor Tyrosine Kinase (RTK)	Receptor Tyrosine Kinase (RTK)	FLT3		FLT3	p.V592D		NantOmics
					KRAS p.G12D	RAS/RAF/MEK				KRAS	p.G12D		NantOmics
							B-cell receptor signaling	BTK					
							PI3K/AKT/mTOR	PIK3CG					
TH01_0130_S01	acute lymphoblastic leukemia	No	Additional expression support for DNA finding	leukemia						new sample			
					PAX5-JAK2 fusion	JAK/STAT				PAX5-JAK2			NantOmics
							Receptor Tyrosine Kinase (RTK)	FLT3					
							B-cell receptor signaling	MS4A1					
							PI3K/AKT/mTOR	PIK3CG					
TH02_0082_S01	diffuse intrinsic pontine glioma	No DNA or RNA targets	No DNA or RNA targets	solid						new sample			
					No druggable mutations		No automated leads			H3F3A	p.K27M		Ashion Analytics
TH02_0083_S01	diffuse intrinsic pontine glioma	Yes	Prioritizing among multiple DNA findings	solid						new sample			
										CRKL	Gain		Ashion Analytics
										H3F3A	p.K27M		Ashion Analytics
					KDR gain	Receptor Tyrosine Kinase (RTK)				KDR	Gain		Ashion Analytics
					KIT gain	Receptor Tyrosine Kinase (RTK)				KIT	Gain		Ashion Analytics
										MAPK1	Gain		Ashion Analytics
					MET gain	Receptor Tyrosine Kinase (RTK)				MET	Gain		Ashion Analytics
					PDGFRA gain	Receptor Tyrosine Kinase (RTK)	Receptor Tyrosine Kinase (RTK)	PDGFRA		PDGFRA	Gain		Ashion Analytics
										TP53	p.G245S		Ashion Analytics
TH02_0084_S01	diffuse intrinsic pontine glioma	No DNA or RNA targets	No DNA or RNA targets	solid						new sample			
					No druggable mutations		No automated leads			TP53	p.A159P		Ashion Analytics
TH02_0085_S01	diffuse intrinsic pontine glioma	Yes	Prioritizing among multiple DNA findings	solid						new sample			
										H3F3A	p.K27M		Ashion Analytics
					KDR gain	Receptor Tyrosine Kinase (RTK)				KDR	Gain		Ashion Analytics
					KIT gain	Receptor Tyrosine Kinase (RTK)				KIT	Gain		Ashion Analytics
					PDGFRA gain	Receptor Tyrosine Kinase (RTK)	Receptor Tyrosine Kinase (RTK)		PDGFRA	PDGFRA	Gain		Ashion Analytics
										TP53	p.A159P		Ashion Analytics
TH02_0086_S01	diffuse intrinsic pontine glioma	RNA only target	RNA only target	solid						new sample			
					No druggable mutations		Sonic Hedgehog (SHH)		PTCH1	ATRX	p.W2001X		Ashion Analytics
										H3F3A	p.K27M		Ashion Analytics
										PPM1D	p.S421X		Ashion Analytics
TH02_0087_S01	diffuse intrinsic pontine glioma	No		solid						new sample			
										H3F3A	p.K27M		Ashion Analytics
					PIK3R1 unspecified aberration, called druggable by clinical testing lab	PI3K/AKT/mTOR				PIK3R1	p.K567E		Ashion Analytics
										PPM1D	p.I546fs		Ashion Analytics
										TP53	p.R282W		Ashion Analytics
TH02_0087_S02	diffuse intrinsic pontine glioma	DNA only target	DNA only target	solid						new sample			
										H3F3A	p.K27M		Ashion Analytics
					PIK3R1 unspecified aberration, called druggable by clinical testing lab	PI3K/AKT/mTOR	No automated leads			PIK3R1	p.K567E		Ashion Analytics
										PPM1D	p.I546fs		Ashion Analytics
TH02_0088_S01	diffuse intrinsic pontine glioma	No DNA or RNA targets	No DNA or RNA targets	solid						new sample			
					No druggable mutations		No automated leads			ATRX	Deletion		Ashion Analytics
										H3F3A	p.K27M		Ashion Analytics
										TP53	p.S37Y		Ashion Analytics
										TP53	p.V157L		Ashion Analytics
TH02_0089_S01	diffuse intrinsic pontine glioma	No DNA or RNA targets	No DNA or RNA targets	solid						new sample			
					No druggable mutations		No automated leads			ATRX	p.A1812P		Ashion Analytics
										H3F3A	p.K27M		Ashion Analytics
										TP53	p.R248W		Ashion Analytics
TH02_0090_S01	diffuse intrinsic pontine glioma	Yes	Additional expression support for DNA finding	solid						new sample			
							Sonic Hedgehog (SHH)		PTCH1				

© 2019 Vaske OM et al. *JAMA Network Open*.

										ATRX	p.E2279X		Ashion Analytics
					IGF1R gain	Receptor Tyrosine Kinase (RTK)				IGF1R	Gain		Ashion Analytics
										TP53	p.R110L		Ashion Analytics
TH02_0091_S01	diffuse intrinsic pontine glioma	No		solid						new sample			
							Receptor Tyrosine Kinase (RTK)	CSF1R					
					BRAF p.V600E	RAS/RAF/MEK				BRAF	p.V600E		Ashion Analytics
										H3F3A	p.K27M		Ashion Analytics
TH02_0092_S01	diffuse intrinsic pontine glioma	No	Additional expression support for DNA finding	solid						new sample			
										ATRX	p.K1357fs		Ashion Analytics
							RAS/RAF/MEK	MEK1		H3F3A	p.K27M		Ashion Analytics
					KDR gain	Receptor Tyrosine Kinase (RTK)				KDR	Gain		Ashion Analytics
					KIT gain	Receptor Tyrosine Kinase (RTK)				KIT	Gain		Ashion Analytics
					PDGFRA gain	Receptor Tyrosine Kinase (RTK)				PDGFRA	Gain		Ashion Analytics
										PTEN	Deletion		Ashion Analytics
										TP53	p.R196P		Ashion Analytics
TH02_0093_S01	diffuse intrinsic pontine glioma	DNA only target	DNA only target	solid						new sample			
							No automated leads			H3F3A	p.K27M		Ashion Analytics
					MET gain	Receptor Tyrosine Kinase (RTK)				MET	Gain		Ashion Analytics
					PDGFRA gain	Receptor Tyrosine Kinase (RTK)				PDGFRA	Gain		Ashion Analytics
										TP53	p.C242Y		Ashion Analytics
										TP53	p.K120E		
TH02_0094_S01	diffuse intrinsic pontine glioma	Yes	Additional expression support for DNA finding	solid						new sample			
										H3F3A	p.K27M		Ashion Analytics
					MET gain	Receptor Tyrosine Kinase (RTK)	Receptor Tyrosine Kinase (RTK)		MET	MET	Gain		Ashion Analytics
										TP53	p.R273C		Ashion Analytics
TH02_0095_S01	diffuse intrinsic pontine glioma	RNA only target	RNA only target	solid						new sample			
					No druggable mutations		Sonic Hedgehog (SHH)		PTCH1	ACVR1	p.R206H		Ashion Analytics
										HIST1H3B	p.K27M		Ashion Analytics
										TP53	p.R248Q		Ashion Analytics
TH02_0096_S01	diffuse intrinsic pontine glioma	No DNA or RNA targets	No DNA or RNA targets	solid						new sample			
					No druggable mutations		No automated leads			DDX11	p.R167T		Ashion Analytics
										PPM1D	p.E405X		Ashion Analytics
										H3F3A	p.K27M		Ashion Analytics
TH03_0003_S01	sarcoma	DNA only target	DNA only target	solid						new sample			
							No automated leads			ATRX	LossExons13-35		Foundation Medicine
					CDKN2A/2B deletion	Cell Cycle				CDKN2A	CDKN2A_loss		Foundation Medicine
										CDKN2B	CDKN2B_loss		Foundation Medicine
										TP53	TruncationExon9		Foundation Medicine
TH03_0003_S03	sarcoma	DNA only target	DNA only target	solid						new sample			
							No automated leads			ATRX	LossExons13-35		Foundation Medicine
					CDKN2A/2B deletion	Cell Cycle				CDKN2A	CDKN2A_loss		Foundation Medicine
										CDKN2B	CDKN2B_loss		Foundation Medicine
										TP53	TruncationExon9		Foundation Medicine
TH03_0003_S04	sarcoma	DNA only target	DNA only target	solid						new sample			
							No automated leads			ATRX	LossExons13-35		Foundation Medicine
					CDKN2A/2B deletion	Cell Cycle				CDKN2A	CDKN2A_loss		Foundation Medicine
										CDKN2B	CDKN2B_loss		Foundation Medicine
										TP53	TruncationExon9		Foundation Medicine
TH03_0004_S02	hepatoblastoma	No DNA or RNA targets	No DNA or RNA targets	solid						new sample			
					No druggable mutations		No automated leads			CTNNB1	p.G34V		Foundation Medicine
										CTNNB1	p.G34V		Foundation Medicine
TH03_0004_S03	hepatoblastoma	No DNA or RNA targets	No DNA or RNA targets	solid						new sample			
					No druggable mutations		No automated leads			CTNNB1	p.G34V		Foundation Medicine
										CTNNB1	p.G34V		Foundation Medicine
TH03_0004_S04	hepatoblastoma	RNA only target	RNA only target	solid						new sample			
					No druggable mutations		JAK/STAT	IL6		CTNNB1	p.G34V		Foundation Medicine
							Heat Shock Proteins	HSP90B1		CTNNB1	p.G34V		Foundation Medicine
TH03_0005_S01	nasopharyngeal carcinoma	No DNA or RNA targets	No DNA or RNA targets	solid						new sample			
					No druggable mutations		No automated leads			CTNNB1	DuplicationExons9-13		Foundation Medicine
										CTNNB1	DuplicationExons9-13		Foundation Medicine
										TP53	p.S241F		Foundation Medicine
										TP53	p.S241F		Foundation Medicine
TH03_0006_S01	rhabdomyosarcoma	RNA only target	RNA only target	solid						new sample			
					No druggable mutations		Heat Shock Proteins		HSP90B1	BRD4	RearrangementExon6		Foundation Medicine
										CREBBP	s894fs*64		Foundation Medicine
										IKZF2	DeletionExon 4		Foundation Medicine
										MYC	Amplification(Equivocal)		Foundation Medicine
										TP53	p.R282W		Foundation Medicine
TH03_0006_S03	rhabdomyosarcoma	No DNA or RNA targets	No DNA or RNA targets	solid						new sample			
					No druggable mutations		No automated leads			BRD4	RearrangementExon6		Foundation Medicine

© 2019 Vaske OM et al. *JAMA Network Open*.

										CREBBP	s894fs*64		Foundation Medicine
										IKZF2	DeletionExon 4		Foundation Medicine
										MYC	Amplification(Equivocal)		Foundation Medicine
										TP53	p.R282W		Foundation Medicine
TH03_0008_S01	neuroblastoma	RNA only target	RNA only target	solid						new sample			
					No druggable mutations		PI3K/AKT/mTOR	RPTOR		ATRX	LossExons2-9		Foundation Medicine
							JAK/STAT	STAT5		CIC	p.R215W		Foundation Medicine
										MYC	SingleCopy		Foundation Medicine
TH03_0010_S01	acute leukemia of ambiguous lineage, CNS1	No	Additional expression support for DNA finding	leukemia						new sample			
							B-cell receptor signaling	BTK		CREBBP	SpliceSite5173-1G>A		Foundation Medicine
							Cell cycle	CDK6					
							JAK/STAT	STAT5A		JAK3	p.A573T		Foundation Medicine
					NRAS p.Q61H	RAS/RAF/MEK				NRAS	p.Q61H		Foundation Medicine
										TP53	p.C135F		Foundation Medicine
TH03_0010_S02	acute leukemia of ambiguous lineage, CNS1	No	Additional expression support for DNA finding	leukemia						new sample			
							B-cell receptor signaling	BTK		CREBBP	SpliceSite5173-1G>A		Foundation Medicine
							BCL2/MDM2	MDM2		JAK3	p.A573T		Foundation Medicine
							JAK/STAT	STAT5A					
					NRAS p.Q61H	RAS/RAF/MEK				NRAS	p.Q61H		Foundation Medicine
										TP53	p.C135F		Foundation Medicine
TH03_0011_S01	acute lymphoblastic leukemia	No	Additional expression support for DNA finding	leukemia						new sample			
							B-cell receptor signaling	BTK		BCOR	p.C1572F		Foundation Medicine
							Cell cycle	CCND3		BCOR	p.C1572F		Foundation Medicine
							Receptor Tyrosine Kinase (RTK)	FLT3		CPS1	p.K1281		Foundation Medicine
							JAK/STAT	IL6R		EXOSC6	p.G10C		Foundation Medicine
										EXOSC6	p.G10C		Foundation Medicine
										MAP3K1	p.S939C		Foundation Medicine
										MAP3K1	p.S939C		Foundation Medicine
										MLL2	p.V4305I		Foundation Medicine
										MLL2	p.V4305I		Foundation Medicine
										MLL3	p.G1582V		Foundation Medicine
										MLL3	p.G1582V		Foundation Medicine
										MLL3	p.L804V		Foundation Medicine
										NCOR2	p.K1405R		Foundation Medicine
										NOTCH1	p.T859M		Foundation Medicine
					NRAS p.Q61K	RAS/RAF/MEK				NRAS	p.Q61K		Foundation Medicine
										NTRK1	p.R744H		Foundation Medicine
										PCLO	p.S4289_S4292del		Foundation Medicine
TH03_0012_S01	wilms tumor	RNA only target	RNA only target	solid						new sample			
					No druggable mutations		PI3K/AKT/mTOR		PIK3R1	ABL2	p.T671M		Foundation Medicine
										ALK	p.E1435K		Foundation Medicine
										AR	p.G457_G468del		Foundation Medicine
										ASXL1	p.G645fs*58		Foundation Medicine
										SPEN	p.S2306del		Foundation Medicine
										TP53	p.C176S		Foundation Medicine
TH03_0012_S02	wilms tumor	RNA only target	RNA only target	solid						new sample			
					No druggable mutations		JAK/STAT	STAT3		ABL2	p.T671M		Foundation Medicine
										ALK	p.E1435K		Foundation Medicine
										AR	p.G457_G468del		Foundation Medicine
										ASXL1	p.G645fs*58		Foundation Medicine
										SPEN	p.S2306del		Foundation Medicine
										TP53	p.C176S		Foundation Medicine
TH03_0016_S01	atypical teratoid/rhabdoid tumor	No	Additional expression support for DNA finding	solid						new sample			
							Receptor Tyrosine Kinase (RTK)	ERBB2		SMARCB1	p.P383fs*4+		Foundation Medicine
							Receptor Tyrosine Kinase (RTK)	FGFR1					
							JAK/STAT	IL6					
					SMARCB1 p.P383fs*4+	Chromatin remodeling							
TH03_0019_S01	fibrolamellar hepatocellular carcinoma	RNA only target	RNA only target	solid						new sample			
					No druggable mutations					FANCG	p.A607T		Foundation Medicine
							Receptor Tyrosine Kinase (RTK)	FLT1					
							Receptor Tyrosine Kinase (RTK)	KDR					
TH03_0114_S01	fibrolamellar hepatocellular carcinoma	RNA only target	RNA only target	solid						new sample			
					No druggable mutations		No automated leads			MLL3	p.C310S		Foundation Medicine
										TERT	promoter 124C>T		Foundation Medicine
TH03_0143_S01	osteosarcoma	RNA only target	RNA only target	solid						new sample			
					No druggable mutations		JAK/STAT	IL6		FANCL	p.S351fs*2		Foundation Medicine
										MLL3	p.Y306*		Foundation Medicine
TH03_0143_S02	osteosarcoma	No DNA or RNA targets	No DNA or RNA targets	solid						new sample			
					No druggable mutations		No automated leads			FANCL	p.S351fs*2		Foundation Medicine
										MLL3	p.Y306*		Foundation Medicine

© 2019 Vaske OM et al. *JAMA Network Open*.

TH03_0149_S01	acute myeloid leukemia	Yes	Additional expression support for DNA finding	leukemia						new sample			
					FLT3-ITD	Receptor Tyrosine Kinase (RTK)	Receptor Tyrosine Kinase (RTK)	FLT3		FLT3	FLT3-ITD Y597-E598ins15		Foundation Medicine
							B-cell receptor signaling	BTK		WT1	p.S381fs*67		Foundation Medicine
TH03_0286_S01	hepatocellular carcinoma	RNA only target	RNA only target	solid						new sample			
					No druggable mutations		Receptor Tyrosine Kinase (RTK)	PDGFRB		CTNNB1	SpliceSite14-59_241+79del366		Foundation Medicine
TH03_0288_S01	neuroblastoma	RNA only target	RNA only target	solid						new sample			
					No druggable mutations		Cell cycle	CDK6		ATRX	LossExons2-12		Foundation Medicine
TH03_0290_S01	glioma	No	No expression evidence for DNA target	solid						new sample			
					BRAF p.V600E	RAS/RAF/MEK				BRAF	p.V600E		Foundation Medicine
							JAK/STAT	IL6		CDKN2A	CDKN2A/B Loss		Foundation Medicine
TH03_0013_S02	acute lymphoblastic leukemia	RNA only target	RNA only target	leukemia						new sample			
					No druggable mutations		Cell cycle	CDK6		t(14;19)	translocation		chromosomal analysis
							PI3K/AKT/mTOR	PIK3CD					
							PI3K/AKT/mTOR	PIK3R5		IGH	possible rearrangement		FISH
TH03_0013_S01	acute lymphoblastic leukemia	RNA only target	RNA only target	leukemia						new sample			
					No druggable mutations		Cell cycle	CDK6					
							PI3K/AKT/mTOR	PIK3CD					
							PI3K/AKT/mTOR	PIK3R5		IGH	possible rearrangement		FISH
TH03_0014_S01	sarcoma	RNA only target	RNA only target	solid						new sample			
					No druggable mutations		JAK/STAT		JAK1	EWSR1	EWSR1-FL1 rearrangement		Foundation Medicine
										FLI1	EWSR1-FL1 rearrangement		Foundation Medicine
TH03_0018_S01	sarcoma	RNA only target	RNA only target	solid						new sample			
					No druggable mutations		Receptor Tyrosine Kinase (RTK)	FLT4		EWSR1	negative for rearrangement		Foundation Medicine
										SS18	negative for rearrangement		Foundation Medicine
										FM performed, no alterations identified	none		Foundation Medicine
TH03_0020_S01	medulloblastoma	RNA only target	RNA only target	solid						new sample			
					No druggable mutations		Sonic Hedgehog (SHH)	PTCH1		MYC	High amplification		FISH
TH03_0112_S01	sarcoma	RNA only target	RNA only target	solid						new sample			
					No druggable mutations		Receptor Tyrosine Kinase (RTK)	ERBB2		SS18	SS18-SSX translocation		Foundation Medicine
							PI3K/AKT/mTOR	MLST8		SSX	SS18-SSX translocation		Foundation Medicine
TH03_0112_S02	sarcoma	RNA only target	RNA only target	solid						new sample			
					No druggable mutations		Sonic Hedgehog (SHH)	GLI1		SS18	SS18-SSX translocation		Foundation Medicine
							Sonic Hedgehog (SHH)	PTCH1		SSX	SS18-SSX translocation		Foundation Medicine
TH03_0113_S01	myoepithelial carcinoma of the liver	No	Additional expression support for DNA finding	solid						new sample			
					INI1 loss of expression	Chromatin remodeling	Receptor Tyrosine Kinase (RTK)	FGFR1		INI1	Loss of expression		IHC
TH03_0115_S01	teratoma	No DNA or RNA targets	No DNA or RNA targets	solid						new sample			
					No druggable mutations		No automated leads			MLL3	C310S		Foundation Medicine
TH03_0141_S01	osteosarcoma	Yes	Prioritizing among multiple DNA findings	solid						new sample			
					CCND3 amplification	Cell Cycle	Cell cycle	CCND3		CSK4	Amplification		Foundation Medicine
							Cell cycle	CDK4		MDM2	Amplification		Foundation Medicine
					MDM2 amplification	BCL2/MDM2	BCL2/MDM2	MDM2		CCND3	Amplification		Foundation Medicine
										FRS2	Amplification		Foundation Medicine
TH03_0144_S01	wilms tumor	RNA only target	RNA only target	solid						new sample			
					No druggable mutations		PI3K/AKT/mTOR	MLST8					
							JAK/STAT	STAT1					Foundation Medicine
TH03_0146_S01	sarcoma	RNA only target	RNA only target	solid						new sample			
					No druggable mutations		Cell cycle		CCNE1	CIC	CIC-DUX4 translocation		FISH
							Receptor Tyrosine Kinase (RTK)		FLT4	DUX4	CIC-DUX4 translocation		FISH
TH03_0296_S01	osteosarcoma	No DNA or RNA targets	No DNA or RNA targets	solid						new sample			
					No druggable mutations		No automated leads			MSH3	Splice site 580-2a>g		Foundation Medicine
TH03_0296_S02	osteosarcoma	RNA only target	RNA only target	solid						new sample			
					No druggable mutations		Receptor Tyrosine Kinase (RTK)		TEK	MSH3	Splice site 580-2a>g		Foundation Medicine
TH06_0645_S01	sarcoma	No	Additional expression support for DNA finding	solid						new sample			
					NF1 stop gained	RAS/RAF/MEK				NF1	stop gained		BC POG DNA results
							Receptor Tyrosine Kinase (RTK)	EGFR		POTEI	missense (benign/tolerated)		BC POG DNA results
							Receptor Tyrosine Kinase (RTK)	ERBB3		MUC17	missense		BC POG DNA results
							Sonic Hedgehog (SHH)	PTCH1		HERC2	deletion/frame shift		BC POG DNA results
										NOTCH2	deletion frame shift		BC POG DNA results
										NCOA3	two codon deletion		BC POG DNA results
										MARCKS	fusion with HERC2		BC POG DNA results
										HERC2	fusion with MARCKS		BC POG DNA results
										ASH1L	65 aa insertion		BC POG DNA results
TH06_0646_S01	neurofibroma	No	Additional expression support for DNA finding	solid						new sample			
					NF1 loss	RAS/RAF/MEK				NF1	loss		BC POG DNA results

© 2019 Vaske OM et al. *JAMA Network Open*.

							Receptor Tyrosine Kinase (RTK)		ERBB3	ACSM5	D400N		BC POG DNA results
							Sonic Hedgehog (SHH)		PTCH1	PRSS3	R255G		BC POG DNA results
										PDE4DIP inversion	inversion		BC POG DNA results
										SSH2	loss		BC POG DNA results
TH06_0647_S01	diffuse intrinsic pontine glioma	Yes	Prioritizing among multiple DNA findings	solid						new sample			
										GSX2	amplification		BC POG DNA results
					KDR amplification	Receptor Tyrosine Kinase (RTK)				KDR	amplification		BC POG DNA results
					PDGFRA amplification	Receptor Tyrosine Kinase (RTK)	Receptor Tyrosine Kinase (RTK)		PDGFRA, PDGFRB	PDGFRA	amplification		BC POG DNA results
										BET1L	homozygous loss		BC POG DNA results
										ELOA3	homozygous loss		BC POG DNA results
										GRID1	homozygous loss		BC POG DNA results
										MYO5B	homozygous loss		BC POG DNA results
										PTPN13	homozygous loss		BC POG DNA results
										PTPN14	gain		BC POG DNA results
										HRNR	missense (present in normal)		BC POG DNA results
										H3F3A	missense		BC POG DNA results
										RYR2	missense		BC POG DNA results
										POMP	missense		BC POG DNA results
										TP53	copy loss		BC POG DNA results
										GOLGA6L2	missense		BC POG DNA results
										TP53	missense		BC POG DNA results
										KRTAP2-2	missense		BC POG DNA results
										SCN4A	missense		BC POG DNA results
										MAST3	missense		BC POG DNA results
										TDRD12	missense		BC POG DNA results
										TDRD12	missense		BC POG DNA results
										UBE2F	missense		BC POG DNA results
										MUC4	missense		BC POG DNA results
										TMEM165	missense		BC POG DNA results
										KHDRBS2	missense		BC POG DNA results
TH06_0648_S01	acute lymphoblastic leukemia	Yes	Prioritizing among multiple DNA findings	leukemia						new sample			
										IGHJ6	translocation		BC POG DNA results
										IGHM	translocation		BC POG DNA results
										KLF9	translocation		BC POG DNA results
										NCOR1	translocation		BC POG DNA results
										NUP160	translocation		BC POG DNA results
										SLC29A1	translocation		BC POG DNA results
										SUZ12	translocation		BC POG DNA results
										ZBTB44	translocation		BC POG DNA results
										ZCCHC7	translocation		BC POG DNA results
										ETV6	deletion/duplication		BC POG DNA results
					CDKN2A homozygous loss	Cell Cycle	Cell cycle	CCND3, CDK6		CDKN2A	homozygous loss		BC POG DNA results
										CNR2	homozygous loss		BC POG DNA results
										CNTNAP3B	homozygous loss		BC POG DNA results
										DMRTA1	homozygous loss		BC POG DNA results
										MTAP	homozygous loss		BC POG DNA results
										PGBD4P5	homozygous loss		BC POG DNA results
										PNRC2	homozygous loss		BC POG DNA results
										RBM17P3	homozygous loss		BC POG DNA results
										TJP2	homozygous loss		BC POG DNA results
										FLT3	T227M		BC POG DNA results
					RAS p.G12A	RAS/RAF/MEK				RAS	G12A		BC POG DNA results
					RAS p.G13D	RAS/RAF/MEK				RAS	G13D		BC POG DNA results
										PCNT	R3232L		BC POG DNA results
										PCNT	R3232R		BC POG DNA results
										NTRK3	Q185K		BC POG DNA results
										KIT V48L	V48L – not characterized		BC POG DNA results
										ZNF521	V574I		BC POG DNA results
										IGF1R	Y203F		BC POG DNA results
							B-cell receptor signaling	BTK					BC POG DNA results
							Receptor Tyrosine Kinase (RTK)	FLT3					BC POG DNA results
TH06_0649_S01	neuroblastoma	Yes	Prioritizing among multiple DNA findings	solid						new sample			
										CST3	duplication		BC POG DNA results
					RPTOR amplification	PI3K/AKT/mTOR	PI3K/AKT/mTOR		RPTOR	RPTOR	amplification		BC POG DNA results

© 2019 Vaske OM et al. *JAMA Network Open*.

B. Comparative RNA-Seq analysis results for all 144 samples.

Sample ID	Activated gene set lead	Outlier support	Pathway support
TH01_0053_S01	New sample	acute myeloid leukemia	
TH01_0053_S01	B-cell receptor signaling	BTK (pc)	KEGG_CYTOKINE_CYTOKINE_RECEPTOR_INTERACTION (pc_up, 6/267)
TH01_0053_S01	Receptor Tyrosine Kinase (RTK)	FLT3 (pc)	REACTOME_IMMUNE_SYSTEM (pc_up, 11/933)
TH01_0054_S01	New sample	acute lymphoblastic leukemia	
TH01_0054_S01	Receptor Tyrosine Kinase (RTK)	FLT3 (pc)	KEGG_CYTOKINE_CYTOKINE_RECEPTOR_INTERACTION (comm_up, 4/267 and pc_up, 7/267 and pd_up, 5/267)
TH01_0054_S01	Receptor Tyrosine Kinase (RTK)	FLT4 (pc)	HALLMARK_KRAS_SIGNALING_UP (pc_up, 5/200)
TH01_0054_S01	B-cell receptor signaling	MS4A1 (pc)	KEGG_HEMATOPOIETIC_CELL_LINEAGE (pc_up, 8/88)
TH01_0055_S01	New sample	glioma	
TH01_0055_S01	N/A	No druggable outliers	N/A
TH01_0061_S01	New sample	germ cell tumor	
TH01_0061_S01	N/A	No druggable outliers	N/A
TH01_0062_S01	New sample	acute lymphoblastic leukemia	
TH01_0062_S01	B-cell receptor signaling	BTK (pc)	BIOCARTA_BCR_PATHWAY (pc_up, 7/37)
TH01_0062_S01	Cell Cycle	CCND3 (pc)	None
TH01_0062_S01	JAK/STAT	IL6R (pc)	KEGG_JAK_STAT_SIGNALING_PATHWAY (pc_up, 10/155)
TH01_0062_S01	B-cell receptor signaling	MS4A1 (pc)	KEGG_HEMATOPOIETIC_CELL_LINEAGE (pc_up, 11/88)
TH01_0062_S01	PI3K/AKT/mTOR	PIK3CD (pc)	PID_PI3KCI_PATHWAY (pc_up, 7/49)
TH01_0062_S01	PI3K/AKT/mTOR	PIK3R5 (pc)	PID_PI3KCI_PATHWAY (pc_up, 7/49)
TH01_0062_S01	JAK/STAT	STAT5B (pc)	KEGG_JAK_STAT_SIGNALING_PATHWAY (pc_up, 10/155)
TH01_0063_S01	New sample	sarcoma	
TH01_0063_S01	JAK/STAT	STAT2 (pc, pd)	None
TH01_0064_S01	New sample	lymphoma	
TH01_0064_S01	B-cell receptor signaling	MS4A1 (pc)	KEGG_HEMATOPOIETIC_CELL_LINEAGE (comm_up, 3/88 and pc_up, 11/88 and pd_up, 3/88)
TH01_0069_S01	New sample	sarcoma	
TH01_0069_S01	N/A	No druggable outliers	N/A
TH01_0120_S01	New sample	acute myeloid leukemia	
TH01_0120_S01	Cell Cycle	CDK6 (pc)	PID_CMYB_PATHWAY (pc_up, 6/84)
TH01_0120_S01	Receptor Tyrosine Kinase (RTK)	KIT (pc)	PID_CMYB_PATHWAY (pc_up, 6/84)
TH01_0121_S01	New sample	acute lymphoblastic leukemia	
TH01_0121_S01	JAK/STAT	IL6R (pc)	KEGG_JAK_STAT_SIGNALING_PATHWAY (pc_up, 13/155)
TH01_0121_S01	JAK/STAT	JAK3 (pc)	KEGG_JAK_STAT_SIGNALING_PATHWAY (pc_up, 13/155)
TH01_0121_S01	B-cell receptor signaling	MS4A1 (pc)	KEGG_HEMATOPOIETIC_CELL_LINEAGE (comm_up, 7/88 and pc_up, 15/88 and pd_up, 8/88)
TH01_0121_S01	PI3K/AKT/mTOR	PIK3CD (pc)	KEGG_PHOSPHATIDYLINOSITOL_SIGNALING_SYSTEM (pc_up, 9/76)
TH01_0121_S01	PI3K/AKT/mTOR	PIK3CG (pc)	KEGG_PHOSPHATIDYLINOSITOL_SIGNALING_SYSTEM (pc_up, 9/76)
TH01_0121_S01	PI3K/AKT/mTOR	PIK3R5 (pc)	KEGG_PHOSPHATIDYLINOSITOL_SIGNALING_SYSTEM (pc_up, 9/76)
TH01_0121_S01	JAK/STAT	STAT5B (pc)	KEGG_JAK_STAT_SIGNALING_PATHWAY (pc_up, 13/155)
TH01_0122_S01	New sample	acute lymphoblastic leukemia	
TH01_0122_S01	Cell Cycle	CDK6 (pc)	None
TH01_0122_S01	B-cell receptor signaling	MS4A1 (pc)	KEGG_HEMATOPOIETIC_CELL_LINEAGE (pc_up, 6/88)
TH01_0122_S01	PI3K/AKT/mTOR	PIK3CD (pc)	KEGG_B_CELL_RECEPTOR_SIGNALING_PATHWAY (pc_up, 6/75)
TH01_0122_S01	PI3K/AKT/mTOR	PIK3CG (pc)	KEGG_B_CELL_RECEPTOR_SIGNALING_PATHWAY (pc_up, 6/75)
TH01_0123_S01	New sample	acute lymphoblastic leukemia	
TH01_0123_S01	Receptor Tyrosine Kinase (RTK)	FLT3 (pc)	KEGG_HEMATOPOIETIC_CELL_LINEAGE (pc_up, 12/88)
TH01_0123_S01	Receptor Tyrosine Kinase (RTK)	FLT4 (pc)	KEGG_HEMATOPOIETIC_CELL_LINEAGE (pc_up, 12/88)
TH01_0123_S01	JAK/STAT	JAK3 (pc)	KEGG_JAK_STAT_SIGNALING_PATHWAY (pc_up, 8/155)
TH01_0123_S01	B-cell receptor signaling	MS4A1 (pc)	KEGG_HEMATOPOIETIC_CELL_LINEAGE (pc_up, 12/88)
TH01_0123_S01	PI3K/AKT/mTOR	PIK3CD (pc)	PID_PI3KCI_PATHWAY (pc_up, 7/49)
TH01_0123_S01	PI3K/AKT/mTOR	PIK3CG (pc)	PID_PI3KCI_PATHWAY (pc_up, 7/49)
TH01_0123_S01	PI3K/AKT/mTOR	RPTOR (pc)	PID_PI3KCI_PATHWAY (pc_up, 7/49)
TH01_0124_S01	New sample	acute lymphoblastic leukemia	
TH01_0124_S01	Receptor Tyrosine Kinase (RTK)	FLT3 (pc)	KEGG_HEMATOPOIETIC_CELL_LINEAGE (pc_up, 4/88)
TH01_0124_S01	PI3K/AKT/mTOR	PIK3CG (pc)	None
TH01_0125_S01	New sample	acute lymphoblastic leukemia	
TH01_0125_S01	B-cell receptor signaling	BTK (pc)	KEGG_B_CELL_RECEPTOR_SIGNALING_PATHWAY (pc_up, 4/75)
TH01_0125_S01	Cell Cycle	CDK6 (pc)	PID_CMYB_PATHWAY (pc_up, 6/84)
TH01_0125_S01	Receptor Tyrosine Kinase (RTK)	FLT3 (pc)	KEGG_HEMATOPOIETIC_CELL_LINEAGE (pc_up, 4/88)
TH01_0125_S01	PI3K/AKT/mTOR	PIK3CG (pc)	KEGG_B_CELL_RECEPTOR_SIGNALING_PATHWAY (pc_up, 4/75)
TH01_0126_S01	New sample	acute lymphoblastic leukemia	
TH01_0126_S01	N/A	No druggable outliers	N/A
TH01_0127_S01	New sample	acute lymphoblastic leukemia	
TH01_0127_S01	B-cell receptor signaling	BTK (pc)	KEGG_B_CELL_RECEPTOR_SIGNALING_PATHWAY (pc_up, 7/75)
TH01_0127_S01	Receptor Tyrosine Kinase (RTK)	FLT3 (pc)	KEGG_HEMATOPOIETIC_CELL_LINEAGE (pc_up, 5/88)
TH01_0127_S01	BCL2/MDM2	MDM2 (pc)	None
TH01_0127_S01	PI3K/AKT/mTOR	PIK3CG (pc)	KEGG_B_CELL_RECEPTOR_SIGNALING_PATHWAY (pc_up, 7/75)

© 2019 Vaske OM et al. *JAMA Network Open*.

Sample ID	Activated gene set lead	Outlier support	Pathway support
TH01_0128_S01	New sample	acute lymphoblastic leukemia	
TH01_0128_S01	JAK/STAT	IL6R (pc)	KEGG_JAK_STAT_SIGNALING_PATHWAY (pc_up, 7/155)
TH01_0128_S01	PI3K/AKT/mTOR	PIK3CD (pc)	PID_PI3KCI_PATHWAY (pc_up, 4/49)
TH01_0128_S01	PI3K/AKT/mTOR	PIK3CG (pc)	PID_PI3KCI_PATHWAY (pc_up, 4/49)
TH01_0128_S01	PI3K/AKT/mTOR	PIK3R5 (pc)	PID_PI3KCI_PATHWAY (pc_up, 4/49)
TH01_0129_S01	New sample	acute lymphoblastic leukemia	
TH01_0129_S01	B-cell receptor signaling	MS4A1 (pc)	KEGG_HEMATOPOIETIC_CELL_LINEAGE (pc_up, 6/88)
TH01_0129_S01	PI3K/AKT/mTOR	PIK3CG (pc)	KEGG_B_CELL_RECEPTOR_SIGNALING_PATHWAY (pc_up, 6/75)
TH01_0130_S01	New sample	acute lymphoblastic leukemia	
TH01_0130_S01	Cell Cycle	CDK6 (pc)	None
TH01_0130_S01	Receptor Tyrosine Kinase (RTK)	FLT3 (pc)	KEGG_HEMATOPOIETIC_CELL_LINEAGE (pc_up, 6/88)
TH01_0130_S01	B-cell receptor signaling	MS4A1 (pc)	KEGG_HEMATOPOIETIC_CELL_LINEAGE (pc_up, 6/88)
TH01_0130_S01	PI3K/AKT/mTOR	PIK3CG (pc)	KEGG_B_CELL_RECEPTOR_SIGNALING_PATHWAY (pc_up, 6/75)
TH01_0131_S01	New sample	acute lymphoblastic leukemia	
TH01_0131_S01	B-cell receptor signaling	BTK (pc)	ST_B_CELL_ANTIGEN_RECEPTOR (pc_up, 3/40)
TH01_0131_S01	Cell Cycle	CDK6 (pc)	PID_CMYB_PATHWAY (pc_up, 4/84)
TH01_0131_S01	Receptor Tyrosine Kinase (RTK)	FLT3 (pc)	KEGG_HEMATOPOIETIC_CELL_LINEAGE (pc_up, 7/88)
TH01_0131_S01	Receptor Tyrosine Kinase (RTK)	FLT4 (pc)	BIOCARTA_VEGF_PATHWAY (pc_up, 3/29)
TH01_0131_S01	PI3K/AKT/mTOR	PIK3CG (pc)	KEGG_B_CELL_RECEPTOR_SIGNALING_PATHWAY (pc_up, 9/75)
TH01_0132_S01	New sample	acute myeloid leukemia	
TH01_0132_S01	B-cell receptor signaling	BTK (pc)	KEGG_B_CELL_RECEPTOR_SIGNALING_PATHWAY (pc_up, 4/75)
TH01_0132_S01	Cell Cycle	CDK6 (pc)	PID_CMYB_PATHWAY (pc_up, 6/84)
TH01_0132_S01	Receptor Tyrosine Kinase (RTK)	FLT3 (pc)	None
TH01_0132_S01	PI3K/AKT/mTOR	PIK3CG (pc)	KEGG_B_CELL_RECEPTOR_SIGNALING_PATHWAY (pc_up, 4/75)
TH01_0133_S01	New sample	acute lymphoblastic leukemia	
TH01_0133_S01	Receptor Tyrosine Kinase (RTK)	FLT3 (pc)	None
TH01_0134_S01	New sample	acute lymphoblastic leukemia	
TH01_0134_S01	Cell Cycle	CDK6 (pc)	PID_CMYB_PATHWAY (pc_up, 9/84)
TH01_0134_S01	Receptor Tyrosine Kinase (RTK)	ERBB2 (pd)	None
TH02_0074_S01	New sample	glioblastoma multiforme	
TH02_0074_S01	PI3K/AKT/mTOR	AKT3 (pc, pd)	PID_PI3KCI_AKT_PATHWAY (comm_up, 3/35)
TH02_0074_S01	Cell Cycle	CCND2 (pc, pd)	KEGG_CELL_CYCLE (comm_up, 6/128 and pc_up, 6/128 and pd_up, 13/128)
TH02_0074_S01	Heat Shock Proteins	HSP90AA1 (pc, pd)	None
TH02_0074_S01	Heat Shock Proteins	HSP90AB1 (pd)	None
TH02_0074_S01	Heat Shock Proteins	HSP90B1 (pd)	HALLMARK_UNFOLDED_PROTEIN_RESPONSE (pd_up, 13/113)
TH02_0074_S01	JAK/STAT	JAK1 (pd)	KEGG_JAK_STAT_SIGNALING_PATHWAY (comm_up, 6/155 and pc_up, 6/155)
TH02_0074_S01	PI3K/AKT/mTOR	PIK3C3 (pd)	None
TH02_0074_S01	PI3K/AKT/mTOR	PIK3R3 (pc, pd)	HALLMARK_MTORC1_SIGNALING (pd_up, 21/200)
TH02_0075_S01	New sample	glioblastoma multiforme	
TH02_0075_S01	PI3K/AKT/mTOR	PIK3R3 (pc, pd)	None
TH02_0076_S01	New sample	glioblastoma multiforme	
TH02_0076_S01	Cell Cycle	CDK4 (pd)	REACTOME_CELL_CYCLE (comm_up, 8/421 and pd_up, 20/421)
TH02_0076_S01	Heat Shock Proteins	HSP90AA1 (pd)	None
TH02_0076_S01	Receptor Tyrosine Kinase (RTK)	MET (pd)	None
TH02_0077_S01	New sample	glioma	
TH02_0077_S01	PI3K/AKT/mTOR	AKT3 (pd)	REACTOME_PI3K_AKT_ACTIVATION (pc_up, 3/38)
TH02_0077_S01	Receptor Tyrosine Kinase (RTK)	PDGFRA (pc)	BIOCARTA_PDGF_PATHWAY (pc_up, 3/32)
TH02_0077_S01	PI3K/AKT/mTOR	PIK3R1 (pc)	REACTOME_PI3K_AKT_ACTIVATION (pc_up, 3/38)
TH02_0077_S01	Sonic Hedgehog (SHH)	PTCH1 (pc)	None
TH02_0078_S01	New sample	glioma	
TH02_0078_S01	PI3K/AKT/mTOR	AKT3 (pd)	KEGG_GLIOMA (comm_up, 3/65 and pc_up, 5/65 and pd_up, 6/65)
TH02_0078_S01	RAS/RAF/MEK	BRAF (pd)	KEGG_GLIOMA (comm_up, 3/65 and pc_up, 5/65 and pd_up, 6/65)
TH02_0078_S01	Cell Cycle	CDK6 (pc, pd)	KEGG_GLIOMA (comm_up, 3/65 and pc_up, 5/65 and pd_up, 6/65)
TH02_0078_S01	Receptor Tyrosine Kinase (RTK)	PDGFRA (pc, pd)	KEGG_GLIOMA (comm_up, 3/65 and pc_up, 5/65 and pd_up, 6/65)
TH02_0078_S01	PI3K/AKT/mTOR	PIK3R1 (pc)	KEGG_GLIOMA (comm_up, 3/65 and pc_up, 5/65 and pd_up, 6/65)
TH02_0079_S01	New sample	glioma	
TH02_0079_S01	N/A	No druggable outliers	N/A
TH02_0080_S01	New sample	glioma	
TH02_0080_S01	N/A	No druggable outliers	N/A
TH02_0081_S01	New sample	glioblastoma multiforme	
TH02_0081_S01	PI3K/AKT/mTOR	AKT3 (pd)	REACTOME_PI_3K_CASCADE (pc_up, 5/56 and pd_up, 6/56)
TH02_0081_S01	BCL2/MDM2	BCL6 (pc)	None
TH02_0081_S01	Cell Cycle	CDK4 (pc, pd)	REACTOME_CELL_CYCLE (pd_up, 17/421)
TH02_0081_S01	PI3K/AKT/mTOR	PIK3C3 (pc, pd)	None
TH02_0081_S01	PI3K/AKT/mTOR	PIK3R1 (pc)	REACTOME_PI_3K_CASCADE (pc_up, 5/56 and pd_up, 6/56)
TH02_0082_S01	New sample	diffuse intrinsic pontine glioma	

© 2019 Vaske OM et al. *JAMA Network Open*.

Sample ID	Activated gene set lead	Outlier support	Pathway support
TH02_0082_S01	N/A	No druggable outliers	N/A
TH02_0083_S01	New sample	diffuse intrinsic pontine glioma	
TH02_0083_S01	Receptor Tyrosine Kinase (RTK)	PDGFRA (pc)	KEGG_MAPK_SIGNALING_PATHWAY (pc_up, 6/267)
TH02_0084_S01	New sample	diffuse intrinsic pontine glioma	
TH02_0084_S01	N/A	No druggable outliers	N/A
TH02_0085_S01	New sample	diffuse intrinsic pontine glioma	
TH02_0085_S01	Receptor Tyrosine Kinase (RTK)	PDGFRA (pc)	None
TH02_0086_S01	New sample	diffuse intrinsic pontine glioma	
TH02_0086_S01	Sonic Hedgehog (SHH)	PTCH1 (pc)	None
TH02_0087_S01	New sample	diffuse intrinsic pontine glioma	
TH02_0087_S01	JAK/STAT	JAK1 (pd)	REACTOME_INTERFERON_SIGNALING (pd_up, 4/159)
TH02_0087_S02	New sample	diffuse intrinsic pontine glioma	
TH02_0087_S02	N/A	No druggable outliers	N/A
TH02_0088_S01	New sample	diffuse intrinsic pontine glioma	
TH02_0088_S01	N/A	No druggable outliers	N/A
TH02_0089_S01	New sample	diffuse intrinsic pontine glioma	
TH02_0089_S01	N/A	No druggable outliers	N/A
TH02_0090_S01	New sample	diffuse intrinsic pontine glioma	
TH02_0090_S01	Sonic Hedgehog (SHH)	PTCH1 (pc)	None
TH02_0091_S01	New sample	diffuse intrinsic pontine glioma	
TH02_0091_S01	Receptor Tyrosine Kinase (RTK)	ALK (pc, pd)	None
TH02_0091_S01	Receptor Tyrosine Kinase (RTK)	CSF1R (pc)	KEGG_HEMATOPOIETIC_CELL_LINEAGE (pc_up, 4/88 and pd_up, 3/88)
TH02_0092_S01	New sample	diffuse intrinsic pontine glioma	
TH02_0092_S01	RAS/RAF/MEK	MAP2K1 (pc, pd)	KEGG_MAPK_SIGNALING_PATHWAY (comm_up, 5/267 and pd_up, 8/267)
TH02_0093_S01	New sample	diffuse intrinsic pontine glioma	
TH02_0093_S01	N/A	No druggable outliers	N/A
TH02_0094_S01	New sample	diffuse intrinsic pontine glioma	
TH02_0094_S01	Receptor Tyrosine Kinase (RTK)	MET (pd)	None
TH02_0095_S01	New sample	diffuse intrinsic pontine glioma	
TH02_0095_S01	Sonic Hedgehog (SHH)	PTCH1 (pc)	None
TH02_0096_S01	New sample	diffuse intrinsic pontine glioma	
TH02_0096_S01	N/A	No druggable outliers	N/A
TH03_0003_S01	New sample	sarcoma	
TH03_0003_S01	N/A	No druggable outliers	N/A
TH03_0003_S03	New sample	sarcoma	
TH03_0003_S03	N/A	No druggable outliers	N/A
TH03_0003_S04	New sample	sarcoma	
TH03_0003_S04	N/A	No druggable outliers	N/A
TH03_0004_S02	New sample	hepatoblastoma	
TH03_0004_S02	N/A	No druggable outliers	N/A
TH03_0004_S03	New sample	hepatoblastoma	
TH03_0004_S03	N/A	No druggable outliers	N/A
TH03_0004_S04	New sample	hepatoblastoma	
TH03_0004_S04	Heat Shock Proteins	HSP90B1 (pd)	HALLMARK_UNFOLDED_PROTEIN_RESPONSE (pd_up, 5/113)
TH03_0004_S04	JAK/STAT	IL6 (pc, pd)	HALLMARK_IL6_JAK_STAT3_SIGNALING (comm_up, 5/87 and pc_up, 5/87 and pd_up, 6/87)
TH03_0005_S01	New sample	nasopharyngeal carcinoma	
TH03_0005_S01	N/A	No druggable outliers	N/A
TH03_0006_S01	New sample	rhabdomyosarcoma	
TH03_0006_S01	Heat Shock Proteins	HSP90B1 (NA)	None
TH03_0006_S03	New sample	rhabdomyosarcoma	
TH03_0006_S03	N/A	No druggable outliers	N/A
TH03_0008_S01	New sample	neuroblastoma	
TH03_0008_S01	PI3K/AKT/mTOR	RPTOR (pc, pd)	HALLMARK_PI3K_AKT_MTOR_SIGNALING (comm_up, 5/105 and pc_up, 7/105 and pd_up, 9/105)
TH03_0008_S01	JAK/STAT	STAT5 (NA)	REACTOME_IMMUNE_SYSTEM (pc_up, 22/933 and pd_up, 31/933)
TH03_0010_S01	New sample	acute leukemia of ambiguous lineage, CNS1	
TH03_0010_S01	B-cell receptor signaling	BTK (pc)	SIG_BCR_SIGNALING_PATHWAY (pc_up, 4/46)
TH03_0010_S01	Cell Cycle	CDK6 (pc)	PID_CMYB_PATHWAY (pc_up, 6/84)
TH03_0010_S01	Receptor Tyrosine Kinase (RTK)	FLT3 (pc)	None
TH03_0010_S01	JAK/STAT	STAT5A (pc)	PID_IL4_2PATHWAY (pc_up, 5/65)
TH03_0010_S02	New sample	acute leukemia of ambiguous lineage, CNS1	
TH03_0010_S02	B-cell receptor signaling	BTK (pc)	REACTOME_SIGNALING_BY_THE_B_CELL_RECEPTOR_BCR (pd_up, 6/126)
TH03_0010_S02	BCL2/MDM2	MDM2 (pd)	HALLMARK_P53_PATHWAY (pd_up, 5/200)
TH03_0010_S02	JAK/STAT	STAT5A (pc)	BIOCARTA_IL3_PATHWAY (pc_up, 2/15)
TH03_0011_S01	New sample	acute lymphoblastic leukemia	

© 2019 Vaske OM et al. *JAMA Network Open*.

Sample ID	Activated gene set lead	Outlier support	Pathway support
TH03_0011_S01	B-cell receptor signaling	BTK (pc)	REACTOME_SIGNALING_BY_THE_B_CELL_RECEPTOR_BCR (pc_up, 10/126)
TH03_0011_S01	Cell Cycle	CCND3 (pc)	BIOCARTA_CELLCYCLE_PATHWAY (pc_up, 3/23)
TH03_0011_S01	Receptor Tyrosine Kinase (RTK)	FLT3 (pc)	KEGG_HEMATOPOIETIC_CELL_LINEAGE (pc_up, 7/88)
TH03_0011_S01	JAK/STAT	IL6R (pc)	KEGG_JAK_STAT_SIGNALING_PATHWAY (pc_up, 6/155)
TH03_0012_S01	New sample	wilms tumor	
TH03_0012_S01	PI3K/AKT/mTOR	PIK3R1 (pd)	None
TH03_0012_S02	New sample	wilms tumor	
TH03_0012_S02	PI3K/AKT/mTOR	PIK3R1 (pd)	None
TH03_0012_S02	JAK/STAT	STAT3 (pd)	HALLMARK_IL6_JAK_STAT3_SIGNALING (pd_up, 7/87)
TH03_0013_S01	New sample	acute lymphoblastic leukemia	
TH03_0013_S01	B-cell receptor signaling	BTK (pc)	REACTOME_SIGNALING_BY_THE_B_CELL_RECEPTOR_BCR (pc_up, 9/126)
TH03_0013_S01	Cell Cycle	CCND3 (pc)	None
TH03_0013_S01	Cell Cycle	CDK6 (pc)	PID_CMYB_PATHWAY (pc_up, 8/84)
TH03_0013_S01	PI3K/AKT/mTOR	PIK3CD (pc)	KEGG_PHOSPHATIDYLINOSITOL_SIGNALING_SYSTEM (pc_up, 4/76)
TH03_0013_S01	PI3K/AKT/mTOR	PIK3R5 (pc)	KEGG_PHOSPHATIDYLINOSITOL_SIGNALING_SYSTEM (pc_up, 4/76)
TH03_0013_S02	New sample	acute lymphoblastic leukemia	
TH03_0013_S02	Cell Cycle	CDK6 (pc)	PID_CMYB_PATHWAY (pc_up, 9/84)
TH03_0013_S02	PI3K/AKT/mTOR	PIK3CD (pc)	KEGG_PHOSPHATIDYLINOSITOL_SIGNALING_SYSTEM (pc_up, 4/76)
TH03_0013_S02	PI3K/AKT/mTOR	PIK3R5 (pc)	KEGG_PHOSPHATIDYLINOSITOL_SIGNALING_SYSTEM (pc_up, 4/76)
TH03_0014_S01	New sample	sarcoma	
TH03_0014_S01	JAK/STAT	JAK1 (pc, pd)	None
TH03_0015_S01	New sample	neuroblastoma	
TH03_0015_S01	Cell Cycle	CDK6 (pc)	REACTOME_CELL_CYCLE (pd_up, 7/421)
TH03_0016_S01	New sample	atypical teratoid/rhabdoid tumor	
TH03_0016_S01	Receptor Tyrosine Kinase (RTK)	ERBB2 (pd)	REACTOME_PI3K_EVENTS_IN_ERBB2_SIGNALING (pd_up, 4/44)
TH03_0016_S01	Receptor Tyrosine Kinase (RTK)	FGFR1 (pd)	PID_FGF_PATHWAY (pd_up, 4/55)
TH03_0016_S01	JAK/STAT	IL6 (pc)	PID_IL6_7_PATHWAY (pc_up, 3/47 and pd_up, 5/47)
TH03_0017_S01	New sample	hepatoblastoma	
TH03_0017_S01	BCL2/MDM2	MDM2 (pc, pd)	KEGG_P53_SIGNALING_PATHWAY (pd_up, 4/69)
TH03_0018_S01	New sample	sarcoma	
TH03_0018_S01	Receptor Tyrosine Kinase (RTK)	FLT4 (pc, pd)	HALLMARK_KRAS_SIGNALING_UP (comm_up, 5/200 and pc_up, 6/200 and pd_up, 6/200)
TH03_0018_S02	New sample	sarcoma	
TH03_0018_S02	N/A	No druggable outliers	N/A
TH03_0019_S01	New sample	fibrolamellar hepatocellular carcinoma	
TH03_0019_S01	Receptor Tyrosine Kinase (RTK)	FLT1 (pd)	PID_VEGF_VEGFR_PATHWAY (pd_up, 2/10)
TH03_0019_S01	Receptor Tyrosine Kinase (RTK)	KDR (pd)	PID_VEGF_VEGFR_PATHWAY (pd_up, 2/10)
TH03_0020_S01	New sample	medulloblastoma	
TH03_0020_S01	Sonic Hedgehog (SHH)	PTCH1 (pc)	PID_HEDGEHOG_GLI_PATHWAY (pc_up, 3/48)
TH03_0022_S01	New sample	osteosarcoma	
TH03_0022_S01	Receptor Tyrosine Kinase (RTK)	FGFR1 (pc)	PID_FGF_PATHWAY (pc_up, 4/55)
TH03_0024_S01	New sample	adrenocortical carcinoma	
TH03_0024_S01	N/A	No druggable outliers	N/A
TH03_0025_S02	New sample	osteosarcoma	
TH03_0025_S02	N/A	No druggable outliers	N/A
TH03_0027_S01	New sample	osteosarcoma	
TH03_0027_S01	N/A	No druggable outliers	N/A
TH03_0029_S01	New sample	osteosarcoma	
TH03_0029_S01	N/A	No druggable outliers	N/A
TH03_0038_S01	New sample	osteosarcoma	
TH03_0038_S01	N/A	No druggable outliers	N/A
TH03_0103_S01	New sample	rhabdomyosarcoma	
TH03_0103_S01	RAS/RAF/MEK	MAP2K2 (pc, pd)	BIOCARTA_BARR_MAPK_PATHWAY (pd_up, 2/12)
TH03_0104_S01	New sample	hepatoblastoma	
TH03_0104_S01	N/A	No druggable outliers	N/A
TH03_0105_S01	New sample	rhabdomyosarcoma	
TH03_0105_S01	N/A	No druggable outliers	N/A
TH03_0106_S01	New sample	sarcoma	
TH03_0106_S01	JAK/STAT	JAK1 (pc)	None
TH03_0107_S01	New sample	hepatoblastoma	
TH03_0107_S01	N/A	No druggable outliers	N/A
TH03_0112_S01	New sample	sarcoma	
TH03_0112_S01	Receptor Tyrosine Kinase (RTK)	ERBB2 (pd)	REACTOME_PI3K_EVENTS_IN_ERBB2_SIGNALING (pd_up, 3/44)
TH03_0112_S01	PI3K/AKT/mTOR	MLST8 (pd)	REACTOME_PI3K_AKT_ACTIVATION (pd_up, 3/38)
TH03_0112_S02	New sample	sarcoma	
TH03_0112_S02	Receptor Tyrosine Kinase (RTK)	ERBB2 (pd)	None
TH03_0112_S02	Sonic Hedgehog (SHH)	GLI1 (pc)	PID_HEDGEHOG_GLI_PATHWAY (pc_up, 3/48)

© 2019 Vaske OM et al. *JAMA Network Open*.

Sample ID	Activated gene set lead	Outlier support	Pathway support
TH03_0112_S02	Sonic Hedgehog (SHH)	PTCH1 (pc)	PID_HEDGEHOG_GLI_PATHWAY (pc_up, 3/48)
TH03_0113_S01	New sample	myoepithelial carcinoma of the liver	
TH03_0113_S01	Receptor Tyrosine Kinase (RTK)	FGFR1 (pc, pd)	PID_FGF_PATHWAY (pd_up, 3/55)
TH03_0113_S02	New sample	myoepithelial carcinoma of the liver	
TH03_0113_S02	Receptor Tyrosine Kinase (RTK)	FGFR1 (pc, pd)	REACTOME_FGFR_LIGAND_BINDING_AND_ACTIVATION (comm_up, 2/22 and pc_up, 2/22)
TH03_0114_S01	New sample	fibrolamellar hepatocellular carcinoma	
TH03_0114_S01	N/A	No druggable outliers	N/A
TH03_0115_S01	New sample	teratoma	
TH03_0115_S01	N/A	No druggable outliers	N/A
TH03_0116_S01	New sample	hepatoblastoma	
TH03_0116_S01	N/A	No druggable outliers	N/A
TH03_0117_S01	New sample	glioma	
TH03_0117_S01	Heat Shock Proteins	HSP90B1 (pd)	None
TH03_0117_S01	Receptor Tyrosine Kinase (RTK)	PDGFRB (pd)	PID_PDGFRB_PATHWAY (pd_up, 9/129)
TH03_0118_S01	New sample	acute myeloid leukemia	
TH03_0118_S01	B-cell receptor signaling	BTK (pc)	KEGG_B_CELL_RECEPTOR_SIGNALING_PATHWAY (pc_up, 4/75)
TH03_0118_S01	Receptor Tyrosine Kinase (RTK)	FLT3 (pc)	KEGG_HEMATOPOIETIC_CELL_LINEAGE (pc_up, 6/88)
TH03_0139_S01	New sample	osteosarcoma	
TH03_0139_S01	Cell Cycle	CCNE1 (pc, pd)	REACTOME_CELL_CYCLE (pd_up, 8/421)
TH03_0140_S01	New sample	osteosarcoma	
TH03_0140_S01	N/A	No druggable outliers	N/A
TH03_0141_S01	New sample	osteosarcoma	
TH03_0141_S01	Cell Cycle	CCND3 (pc, pd)	REACTOME_CELL_CYCLE (pc_up, 6/421 and pd_up, 5/421)
TH03_0141_S01	Cell Cycle	CDK4 (pc, pd)	REACTOME_CELL_CYCLE (pc_up, 6/421 and pd_up, 5/421)
TH03_0141_S01	BCL2/MDM2	MDM2 (pd)	BIOCARTA_P53_PATHWAY (pd_up, 2/16)
TH03_0141_S01	Sonic Hedgehog (SHH)	PTCH1 (pc)	None
TH03_0143_S01	New sample	osteosarcoma	
TH03_0143_S01	JAK/STAT	IL6 (pc, pd)	BIOCARTA_IL6_PATHWAY (pd_up, 2/22)
TH03_0143_S02	New sample	osteosarcoma	
TH03_0143_S02	N/A	No druggable outliers	N/A
TH03_0144_S01	New sample	wilms tumor	
TH03_0144_S01	PI3K/AKT/mTOR	MLST8 (pd)	KEGG_MTOR_SIGNALING_PATHWAY (pd_up, 3/52)
TH03_0144_S01	JAK/STAT	STAT1 (pd)	REACTOME_CYTOKINE_SIGNALING_IN_IMMUNE_SYSTEM (pd_up, 11/270)
TH03_0146_S01	New sample	sarcoma	
TH03_0146_S01	Cell Cycle	CCNE1 (pc)	None
TH03_0146_S01	Receptor Tyrosine Kinase (RTK)	FLT4 (pc, pd)	None
TH03_0148_S01	New sample	lymphoma	
TH03_0148_S01	B-cell receptor signaling	MS4A1 (pc, pd)	KEGG_HEMATOPOIETIC_CELL_LINEAGE (comm_up, 8/88 and pc_up, 8/88 and pd_up, 8/88)
TH03_0149_S01	New sample	acute myeloid leukemia	
TH03_0149_S01	Receptor Tyrosine Kinase (RTK)	FLT3 (pc)	KEGG_HEMATOPOIETIC_CELL_LINEAGE (pc_up, 8/88)
TH03_0156_S01	New sample	sarcoma	
TH03_0156_S01	N/A	No druggable outliers	N/A
TH03_0159_S01	New sample	osteosarcoma	
TH03_0159_S01	N/A	No druggable outliers	N/A
TH03_0159_S02	New sample	osteosarcoma	
TH03_0159_S02	N/A	No druggable outliers	N/A
TH03_0285_S01	New sample	juvenile myelomonocytic leukemia	
TH03_0285_S01	B-cell receptor signaling	BTK (pc)	REACTOME_SIGNALING_BY_THE_B_CELL_RECEPTOR_BCR (pc_up, 9/126)
TH03_0285_S01	Receptor Tyrosine Kinase (RTK)	CSF1R (pc)	KEGG_CYTOKINE_CYTOKINE_RECEPTOR_INTERACTION (pc_up, 17/267 and pd_up, 6/267)
TH03_0285_S01	JAK/STAT	IL6 (pd)	KEGG_CYTOKINE_CYTOKINE_RECEPTOR_INTERACTION (pc_up, 17/267 and pd_up, 6/267)
TH03_0285_S01	B-cell receptor signaling	MS4A1 (pc)	KEGG_HEMATOPOIETIC_CELL_LINEAGE (comm_up, 3/88 and pc_up, 15/88 and pd_up, 4/88)
TH03_0286_S01	New sample	hepatocellular carcinoma	
TH03_0286_S01	Receptor Tyrosine Kinase (RTK)	PDGFRB (pd)	KEGG_MAPK_SIGNALING_PATHWAY (pd_up, 4/267)
TH03_0287_S01	New sample	neuroblastoma	
TH03_0287_S01	PI3K/AKT/mTOR	AKT3 (pd)	PID_PI3KCI_AKT_PATHWAY (pd_up, 3/35)
TH03_0288_S01	New sample	neuroblastoma	
TH03_0288_S01	Receptor Tyrosine Kinase (RTK)	ALK (pc)	None
TH03_0288_S01	Cell Cycle	CDK6 (pc)	BIOCARTA_CELLCYCLE_PATHWAY (pc_up, 3/23)
TH03_0288_S01	B-cell receptor signaling	MS4A1 (pc, pd)	None
TH03_0289_S01	New sample	acute lymphoblastic leukemia	
TH03_0289_S01	B-cell receptor signaling	BTK (pc)	KEGG_B_CELL_RECEPTOR_SIGNALING_PATHWAY (pc_up, 11/75)
TH03_0289_S01	Cell Cycle	CCND3 (pc)	REACTOME_CELL_CYCLE (pc_up, 10/421)
TH03_0289_S01	Receptor Tyrosine Kinase (RTK)	FLT3 (pc)	KEGG_HEMATOPOIETIC_CELL_LINEAGE (pc_up, 11/88)
TH03_0289_S01	BCL2/MDM2	MDM2 (pc)	HALLMARK_P53_PATHWAY (pc_up, 6/200)
TH03_0289_S01	B-cell receptor signaling	MS4A1 (pc)	KEGG_HEMATOPOIETIC_CELL_LINEAGE (pc_up, 11/88)
TH03_0289_S01	PI3K/AKT/mTOR	PIK3C2B (pc)	KEGG_PHOSPHATIDYLINOSITOL_SIGNALING_SYSTEM (pc_up, 5/76)

© 2019 Vaske OM et al. *JAMA Network Open*.

Sample ID	Activated gene set lead	Outlier support	Pathway support
TH03_0289_S01	PI3K/AKT/mTOR	PIK3CD (pc)	KEGG_PHOSPHATIDYLINOSITOL_SIGNALING_SYSTEM (pc_up, 5/76)
TH03_0290_S01	New sample	glioma	
TH03_0290_S01	JAK/STAT	IL6 (pd)	HALLMARK_INFLAMMATORY_RESPONSE (pd_up, 5/200)
TH03_0291_S01	New sample	ependymoma	
TH03_0291_S01	Receptor Tyrosine Kinase (RTK)	ERBB2 (pd)	None
TH03_0296_S01	New sample	osteosarcoma	
TH03_0296_S01	N/A	No druggable outliers	N/A
TH03_0296_S02	New sample	osteosarcoma	
TH03_0296_S02	Receptor Tyrosine Kinase (RTK)	TEK (pc, pd)	None
TH06_0284_S01	New sample	neuroblastoma	
TH06_0284_S01	Receptor Tyrosine Kinase (RTK)	ERBB3 (pd)	None
TH06_0610_S01	New sample	sarcoma	
TH06_0610_S01	Receptor Tyrosine Kinase (RTK)	ALK (pc, pd)	
TH06_0610_S01	Cell Cycle	CCND1 (pd)	PID_CMYB_PATHWAY (pd_up, 5/84)
TH06_0610_S01	Receptor Tyrosine Kinase (RTK)	FGFR1 (pd)	PID_FGF_PATHWAY (pd_up, 5/55)
TH06_0610_S01	JAK/STAT	IL6 (pc, pd)	BIOCARTA_IL10_PATHWAY (pd_up, 3/17)
TH06_0610_S01	JAK/STAT	JAK1 (pd)	BIOCARTA_IL10_PATHWAY (pd_up, 3/17)
TH06_0610_S01	JAK/STAT	STAT5B (pd)	BIOCARTA_IL10_PATHWAY (pd_up, 3/17)
TH06_0611_S01	New sample	craniopharyngioma	
TH06_0611_S01	Sonic Hedgehog (SHH)	PTCH1 (pc)	BIOCARTA_SHH_PATHWAY (pc_up, 3/16)
TH06_0612_S01	New sample	thyroid carcinoma	
TH06_0612_S01	N/A	No druggable outliers	N/A
TH06_0613_S01	New sample	atypical teratoid/rhabdoid tumor	
TH06_0613_S01	Cell Cycle	CCND3 (pd)	HALLMARK_P53_PATHWAY (pd_up, 6/200)
TH06_0613_S01	RAS/RAF/MEK	MAP2K1 (pc, pd)	BIOCARTA_ERK_PATHWAY (comm_up, 2/28 and pc_up, 2/28 and pd_up, 3/28)
TH06_0613_S01	RAS/RAF/MEK	MAP2K2 (pc, pd)	BIOCARTA_ERK_PATHWAY (comm_up, 2/28 and pc_up, 2/28 and pd_up, 3/28)
TH06_0613_S02	New sample	atypical teratoid/rhabdoid tumor	
TH06_0613_S02	Receptor Tyrosine Kinase (RTK)	PDGFRA (pd)	None
TH06_0615_S01	New sample	sarcoma	
TH06_0615_S01	Receptor Tyrosine Kinase (RTK)	KIT (pc)	None
TH06_0616_S01	New sample	glioblastoma multiforme	
TH06_0616_S01	PI3K/AKT/mTOR	AKT1 (pd)	HALLMARK_PI3K_AKT_MTOR_SIGNALING (pd_up, 4/105)
TH06_0616_S01	DNA repair	PARP1 (pd)	None
TH06_0616_S01	PI3K/AKT/mTOR	PIK3R2 (pc, pd)	REACTOME_PI3K_AKT_ACTIVATION (pd_up, 3/38)
TH06_0617_S01	New sample	ependymoma	
TH06_0617_S01	PI3K/AKT/mTOR	PIK3R1 (pc, pd)	None
TH06_0618_S01	New sample	NUT midline carcinoma	
TH06_0618_S01	N/A	No druggable outliers	N/A
TH06_0619_S01	New sample	angiosarcoma	
TH06_0619_S01	Receptor Tyrosine Kinase (RTK)	FGFR1 (pd)	REACTOME_SIGNALING_BY_FGFR_IN_DISEASE (pd_up, 5/127)
TH06_0619_S01	Receptor Tyrosine Kinase (RTK)	TEK (pc)	PID_SHP2_PATHWAY (pc_up, 4/58)
TH06_0620_S01	New sample	gliomatosis cerebri	
TH06_0620_S01	N/A	No druggable outliers	N/A
TH06_0621_S01	New sample	osteosarcoma	
TH06_0621_S01	PI3K/AKT/mTOR	MLST8 (pd)	REACTOME_MTORC1_MEDIATED_SIGNALLING (pd_up, 2/11)
TH06_0622_S01	New sample	neuroblastoma	
TH06_0622_S01	N/A	No druggable outliers	N/A
TH06_0623_S01	New sample	rhabdomyosarcoma	
TH06_0623_S01	Receptor Tyrosine Kinase (RTK)	ALK (pc)	None
TH06_0623_S01	Heat Shock Proteins	HSP90AA1 (pc, pd)	None
TH06_0624_S01	New sample	lung adenocarcinoma	
TH06_0624_S01	N/A	No druggable outliers	N/A
TH06_0625_S01	New sample	fibromatosis	
TH06_0625_S01	N/A	No druggable outliers	N/A
TH06_0626_S01	New sample	fibromatosis	
TH06_0626_S01	N/A	No druggable outliers	N/A
TH06_0627_S01	New sample	glioblastoma multiforme	
TH06_0627_S01	PI3K/AKT/mTOR	AKT1 (pd)	PID_PI3KCI_AKT_PATHWAY (pd_up, 3/35)
TH06_0627_S01	BCL2/MDM2	BCL6 (pc)	None
TH06_0627_S01	Receptor Tyrosine Kinase (RTK)	CSF1R (pc)	None
TH06_0627_S01	JAK/STAT	STAT1 (pd)	KEGG_JAK_STAT_SIGNALING_PATHWAY (pd_up, 5/155)
TH06_0627_S01	JAK/STAT	STAT2 (pc, pd)	KEGG_JAK_STAT_SIGNALING_PATHWAY (pd_up, 5/155)
TH06_0628_S01	New sample	neuroblastoma	
TH06_0628_S01	Receptor Tyrosine Kinase (RTK)	CSF1R (pd)	PID_CMYB_PATHWAY (pd_up, 7/84)
TH06_0629_S01	New sample	neurofibromatosis type 1	
TH06_0629_S01	Receptor Tyrosine Kinase (RTK)	ERBB3 (pd)	REACTOME_SIGNALING_BY_ERBB2 (pd_up, 5/101)

© 2019 Vaske OM et al. *JAMA Network Open*.

Sample ID	Activated gene set lead	Outlier support	Pathway support
TH06_0630_S01	New sample	osteosarcoma	
TH06_0630_S01	PI3K/AKT/mTOR	MLST8 (pd)	KEGG_MTOR_SIGNALING_PATHWAY (pd_up, 8/52)
TH06_0630_S01	PI3K/AKT/mTOR	PIK3R1 (pd)	KEGG_MTOR_SIGNALING_PATHWAY (pd_up, 8/52)
TH06_0630_S01	JAK/STAT	STAT2 (pd)	REACTOME_CYTOKINE_SIGNALING_IN_IMMUNE_SYSTEM (pd_up, 12/270)
TH06_0630_S01	PI3K/AKT/mTOR	TSC2 (pc, pd)	KEGG_MTOR_SIGNALING_PATHWAY (pd_up, 8/52)
TH06_0631_S01	New sample	thyroid carcinoma	
TH06_0631_S01	Receptor Tyrosine Kinase (RTK)	CSF1R (pd)	KEGG_HEMATOPOIETIC_CELL_LINEAGE (pd_up, 5/88)
TH06_0631_S01	JAK/STAT	STAT2 (pd)	REACTOME_CYTOKINE_SIGNALING_IN_IMMUNE_SYSTEM (pd_up, 13/270)
TH06_0631_S01	PI3K/AKT/mTOR	TSC2 (pd)	HALLMARK_PI3K_AKT_MTOR_SIGNALING (pd_up, 6/105)
TH06_0632_S01	New sample	wilms tumor	
TH06_0632_S01	BCL2/MDM2	BCL6 (pc, pd)	PID_P53_DOWNSTREAM_PATHWAY (pd_up, 10/137)
TH06_0632_S01	PI3K/AKT/mTOR	MLST8 (pd)	KEGG_MTOR_SIGNALING_PATHWAY (pd_up, 6/52)
TH06_0632_S01	Receptor Tyrosine Kinase (RTK)	PDGFRB (pd)	KEGG_MTOR_SIGNALING_PATHWAY (pd_up, 6/52)
TH06_0632_S01	PI3K/AKT/mTOR	PIK3R1 (pd)	KEGG_MTOR_SIGNALING_PATHWAY (pd_up, 6/52)
TH06_0632_S01	JAK/STAT	STAT3 (pd)	HALLMARK_INTERFERON_GAMMA_RESPONSE (pd_up, 15/200)
TH06_0632_S01	PI3K/AKT/mTOR	TSC2 (pc)	KEGG_MTOR_SIGNALING_PATHWAY (pd_up, 6/52)
TH06_0633_S01	New sample	neurofibroma	
TH06_0633_S01	BCL2/MDM2	BCL6 (pd)	PID_P53_DOWNSTREAM_PATHWAY (pd_up, 14/137)
TH06_0633_S01	Receptor Tyrosine Kinase (RTK)	EGFR (pd)	KEGG_MAPK_SIGNALING_PATHWAY (comm_up, 11/267 and pc_up, 11/267 and pd_up, 23/267)
TH06_0633_S01	Receptor Tyrosine Kinase (RTK)	ERBB2 (pd)	REACTOME_SIGNALING_BY_ERBB2 (comm_up, 4/101 and pc_up, 4/101 and pd_up, 14/101)
TH06_0633_S01	Receptor Tyrosine Kinase (RTK)	FGFR1 (pd)	KEGG_MAPK_SIGNALING_PATHWAY (comm_up, 11/267 and pc_up, 11/267 and pd_up, 23/267)
TH06_0633_S01	Sonic Hedgehog (SHH)	GLI1 (pc, pd)	PID_HEDGEHOG_GLI_PATHWAY (comm_up, 3/48 and pc_up, 3/48 and pd_up, 7/48)
TH06_0633_S01	Receptor Tyrosine Kinase (RTK)	PDGFRB (pc, pd)	PID_PDGFRB_PATHWAY (comm_up, 6/129 and pc_up, 6/129 and pd_up, 14/129)
TH06_0633_S01	PI3K/AKT/mTOR	PIK3R1 (pd)	REACTOME_PI3K_EVENTS_IN_ERBB2_SIGNALING (pd_up, 7/44)
TH06_0633_S01	Sonic Hedgehog (SHH)	PTCH1 (pc, pd)	PID_HEDGEHOG_GLI_PATHWAY (comm_up, 3/48 and pc_up, 3/48 and pd_up, 7/48)
TH06_0633_S01	RAS/RAF/MEK	RAF1 (pd)	KEGG_MAPK_SIGNALING_PATHWAY (comm_up, 11/267 and pc_up, 11/267 and pd_up, 23/267)
TH06_0633_S01	JAK/STAT	STAT3 (pd)	REACTOME_IMMUNE_SYSTEM (pd_up, 40/933)
TH06_0633_S01	JAK/STAT	STAT5B (pd)	REACTOME_IMMUNE_SYSTEM (pd_up, 40/933)
TH06_0633_S01	PI3K/AKT/mTOR	TSC2 (pd)	REACTOME_PI3K_EVENTS_IN_ERBB2_SIGNALING (pd_up, 7/44)
TH06_0634_S01	New sample	osteosarcoma	
TH06_0634_S01	Cell Cycle	CDK4 (pd)	None
TH06_0645_S01	New sample	sarcoma	
TH06_0645_S01	Receptor Tyrosine Kinase (RTK)	EGFR (pd)	REACTOME_SIGNALING_BY_ERBB4 (pd_up, 4/90)
TH06_0645_S01	Receptor Tyrosine Kinase (RTK)	ERBB3 (pd)	REACTOME_SIGNALING_BY_ERBB4 (pd_up, 4/90)
TH06_0645_S01	Sonic Hedgehog (SHH)	PTCH1 (pc, pd)	KEGG_BASAL_CELL_CARCINOMA (comm_up, 3/55 and pc_up, 3/55 and pd_up, 3/55)
TH06_0646_S01	New sample	neurofibroma	
TH06_0646_S01	Receptor Tyrosine Kinase (RTK)	ERBB3 (pd)	None
TH06_0646_S01	Sonic Hedgehog (SHH)	PTCH1 (pc, pd)	None
TH06_0647_S01	New sample	diffuse intrinsic pontine glioma	
TH06_0647_S01	Receptor Tyrosine Kinase (RTK)	PDGFRA (pc)	None
TH06_0647_S01	Receptor Tyrosine Kinase (RTK)	PDGFRB (pd)	None
TH06_0648_S01	New sample	acute lymphoblastic leukemia	
TH06_0648_S01	B-cell receptor signaling	BTK (pc)	REACTOME_SIGNALING_BY_THE_B_CELL_RECEPTOR_BCR (pc_up, 11/126)
TH06_0648_S01	Cell Cycle	CCND3 (pc)	REACTOME_G1_PHASE (pc_up, 3/38)
TH06_0648_S01	Cell Cycle	CDK6 (pc)	REACTOME_G1_PHASE (pc_up, 3/38)
TH06_0648_S01	Receptor Tyrosine Kinase (RTK)	FLT3 (pc)	KEGG_HEMATOPOIETIC_CELL_LINEAGE (pc_up, 7/88)
TH06_0649_S01	New sample	neuroblastoma	
TH06_0649_S01	PI3K/AKT/mTOR	RPTOR (pc, pd)	None

© 2019 Vaske OM et al. *JAMA Network Open*.

a.

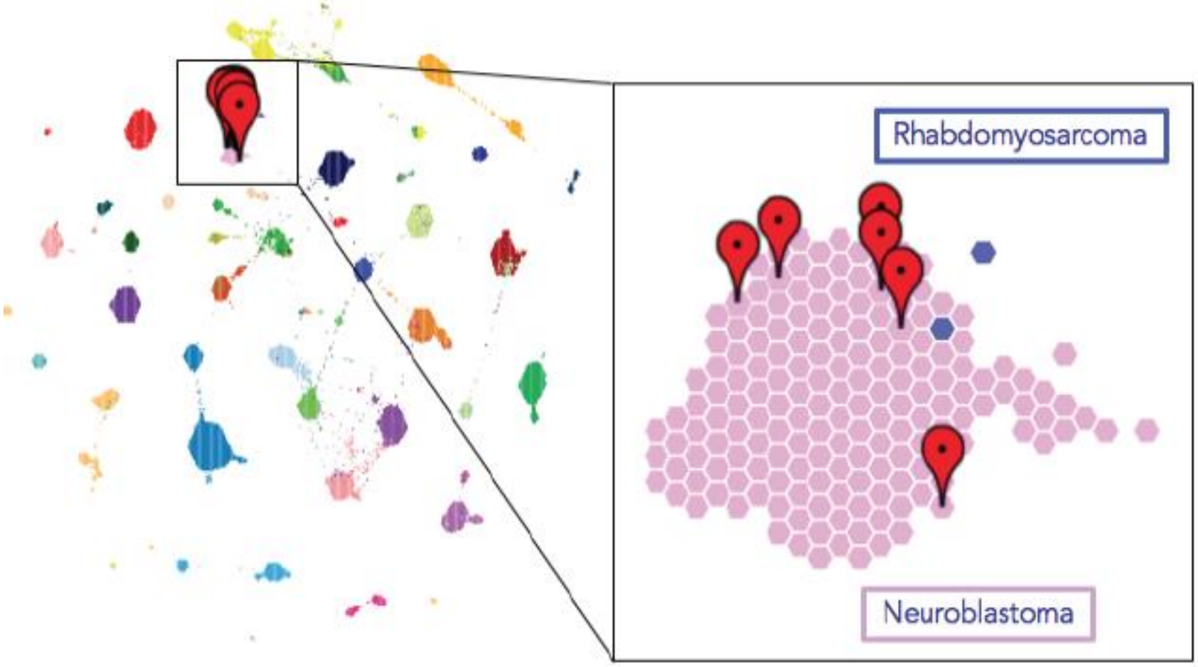

b.

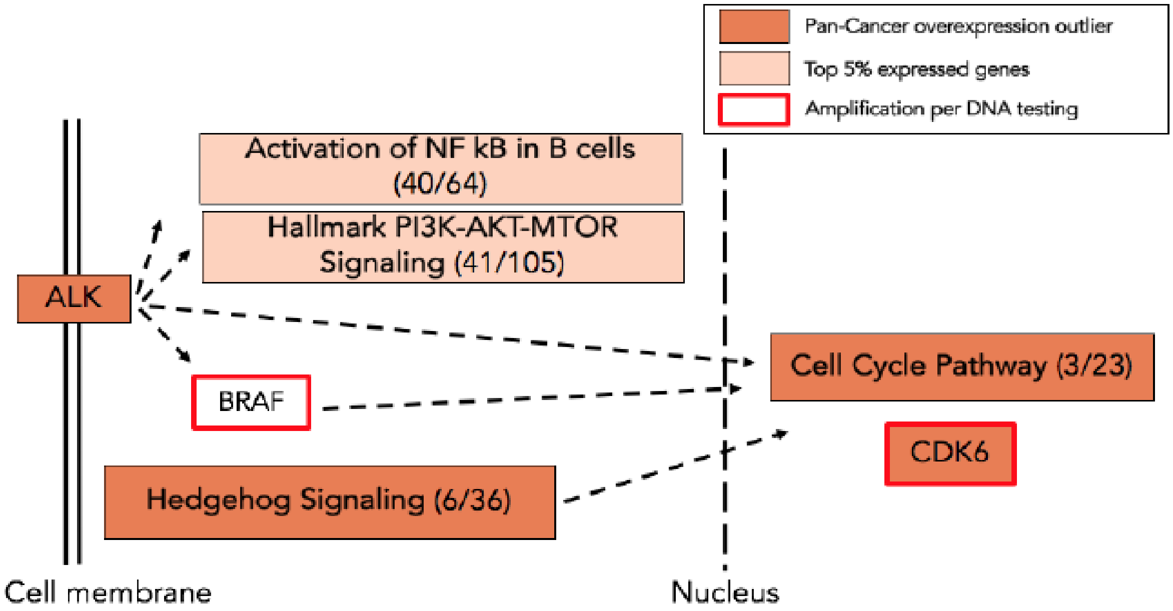

© 2019 Vaske OM et al. *JAMA Network Open*.

c.

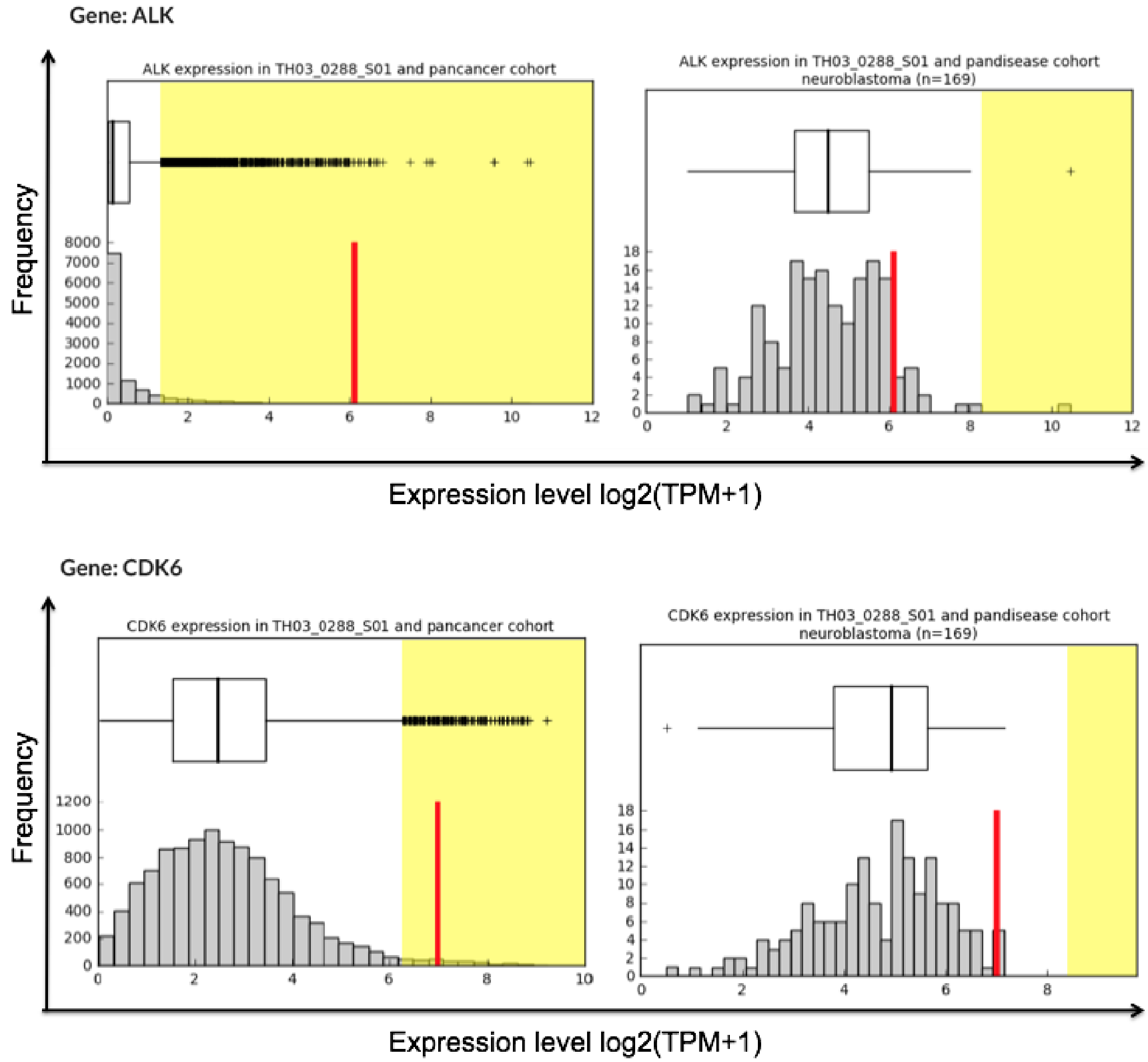

eFigure 2. Treehouse analysis of sample TH03_0288_S01. A. Gene expression similarity analysis, visualized using TumorMap (eMethods), reveals that most correlated samples are neuroblastomas. B. Summary of outlier analysis and gene set overlap analysis of lists of outlier genes. ALK and CDK6 are pan-cancer over-expression outliers; CDK6 and BRAF are amplified in the tumor. The Hedgehog Signaling and Cell Cycle Pathway annotations are significantly enriched among outlier gene lists. C. Gene expression levels of ALK (top) and CDK6 (bottom) in the whole compendium (left) or all neuroblastoma samples (right). The outlier range is denoted in yellow, and the expression level in sample TH03_0288_S01 is indicated with a red bar.

© 2019 Vaske OM et al. *JAMA Network Open*.

eReferences

1. Vivian J, Rao AA, Nothaft FA, et al. Toil enables reproducible, open source, big biomedical data analyses. *Nat Biotechnol*. 2017;35(4):314-316. doi:10.1038/nbt.3772

2. Harris PA, Taylor R, Thielke R, Payne J, Gonzalez N, Conde JG. Research electronic data capture (REDCap)--a metadata-driven methodology and workflow process for providing translational research informatics support. *J Biomed Inform*. 2009;42(2):377-381. doi:10.1016/j.jbi.2008.08.010

3. Newton Y, Novak AM, Swatloski T, et al. TumorMap: Exploring the Molecular Similarities of Cancer Samples in an Interactive Portal. *Cancer Res*. 2017;77(21):e111-e114. doi:10.1158/0008-5472.CAN-17-0580

© 2019 Vaske OM et al. *JAMA Network Open*.
